# Intravenous chaperone treatment of late-stage Alzheimer´s disease (AD) mouse model affects amyloid plaque load, reactive gliosis and AD-related genes

**DOI:** 10.1038/s41398-024-03161-x

**Published:** 2024-10-24

**Authors:** Ruixin Zhang, Makiko Ohshima, David Brodin, Yu Wang, Antonin Morancé, Marianne Schultzberg, Gefei Chen, Jan Johansson

**Affiliations:** 1 Department of Medicine Huddinge, Karolinska Institutet, Huddinge, Sweden; 2 Division of Neurogeriatrics, Department of Neurobiology, Care Sciences & Society, Karolinska Institutet, Solna, Sweden; 3 Department of Neuroscience, University of Mons (UMONS), Mons, Belgium; 4 Department of Cell and Molecular Biology, Uppsala University, Uppsala, Sweden

**Keywords:** Molecular neuroscience, Diseases

## Abstract

Treatment strategies that are efficient against established Alzheimer’s disease (AD) are needed. BRICHOS is a molecular chaperone domain that prevents amyloid fibril formation and associated cellular toxicity. In this study, we treated an AD mouse model seven months after pathology onset, using intravenous administration of recombinant human (rh) Bri2 BRICHOS R221E. Two injections of rh Bri2 BRICHOS R221E per week for three months in AD mice reduced amyloid β (Aβ) burden, and mitigated astro- and microgliosis, as determined by glial fibrillary acidic protein (GFAP) and ionized calcium-binding adaptor molecule 1 (Iba1) immunohistochemistry. Sequencing of RNA from cortical microglia cells showed that BRICHOS treatment normalized the expression of identified plaque-induced genes in mice and humans, including clusterin and GFAP. Rh Bri2 BRICHOS R221E passed the blood–brain barrier (BBB) in age-matched wild-type mice as efficiently as in the AD mice, but then had no effect on measures of AD-like pathology, and mainly affected the expression of genes that affect cellular shape and movement. These results indicate a potential of rh Bri2 BRICHOS against advanced AD and underscore the ability of BRICHOS to target amyloid-induced pathology.

## Introduction

Alzheimer’s disease (AD), the most common form of dementia, poses significant medical and socioeconomic problems [1]. The rapidly increasing number of patients afflicted by this ailment underscores the urgent need for the development of efficacious treatments. Recently, antibody-based drugs targeting the amyloid cascade have undergone clinical trials for early symptomatic AD, yielding varied levels of success [2]. Notably, the monoclonal antibodies Aducanumab and Lecanemab, targeting amyloid-β (Aβ) fibrillar assemblies, one hallmark of AD, have been approved by the U.S. Food and Drug Administration (FDA) for treating individuals in the early symptomatic stages of AD. The monoclonal antibody Donanemab, known to bind to amyloid plaques, decelerates memory and cognitive decline in individuals with early-stage AD [3]. While these antibody drugs mitigate memory and cognitive deterioration in early-stage AD, their efficacies are limited, and side effects have been reported [4, 5]. AD is typically diagnosed in its later stages [6], indicating the need for candidate drugs that can affect established AD pathology. AD is characterized by the accumulation of extracellular Aβ plaques and intraneuronal formation of tau neurofibrillary tangles [7, 8]. The Aβ peptide is produced through successive cleavages of amyloid-β precursor protein (APP) by β-secretase 1 (BACE1) and γ-secretase, and AD pathogenesis is coupled to the accumulation of extracellular Aβ, in particular Aβ_42_, in cortical and subcortical brain regions [9]. AD pathology also includes activation of microglia and astrocytes, and increased levels of proinflammatory mediators [10, 11].

Molecular chaperones play an important role in maintaining cellular proteostasis, and can prevent or resolve protein aggregation [12]. The BRICHOS domain was initially identified in Bri2, chondromodulin-1, and prosurfactant protein C (proSP-C). Each of these proproteins contain a region prone to form amyloid [13, 14], which BRICHOS chaperones during biosynthesis [15]. Furthermore, recombinant human (rh) BRICHOS domains from proSP-C and Bri2 are potent inhibitors of amyloid formation also by non-client proteins such as medin, islet amyloid polypeptide (IAPP), Aβ_40_ and Aβ_42_ [16, 17]. Bri2 is highly expressed in neurons of the human hippocampus and cortex, co-localizes with AD senile plaques [18, 19] and releases the BRICHOS domain [20]. Rh Bri2 BRICHOS potently inhibits Aβ_42_ fibril formation and toxicity in vitro, in hippocampal slice preparations, as well as in vivo in *Drosophila melanogaster* and AD mouse models [21–24]. The Bri2 BRICHOS monomer is most effective in preventing Aβ_42_-induced disruption of neuronal network activity [25], and efficiently crosses the blood–brain barrier (BBB) [26]. Therefore, a Bri2 BRICHOS mutant, R221E, which forms long-term stable monomers has been designed [27]. Rh Bri2 BRICHOS R221E administered intravenously (i.v.) to *App* knock-in mouse models improved recognition and working memory, reduced Aβ plaque deposition and mitigated activation of astrocytes and microglia [24]. However, it remains unclear whether BRICHOS treatment is effective when applied at advanced AD-like pathology stages, and if BRICHOS can normalize Aβ-induced dysregulation of AD-associated genes.

**Fig. 1:**
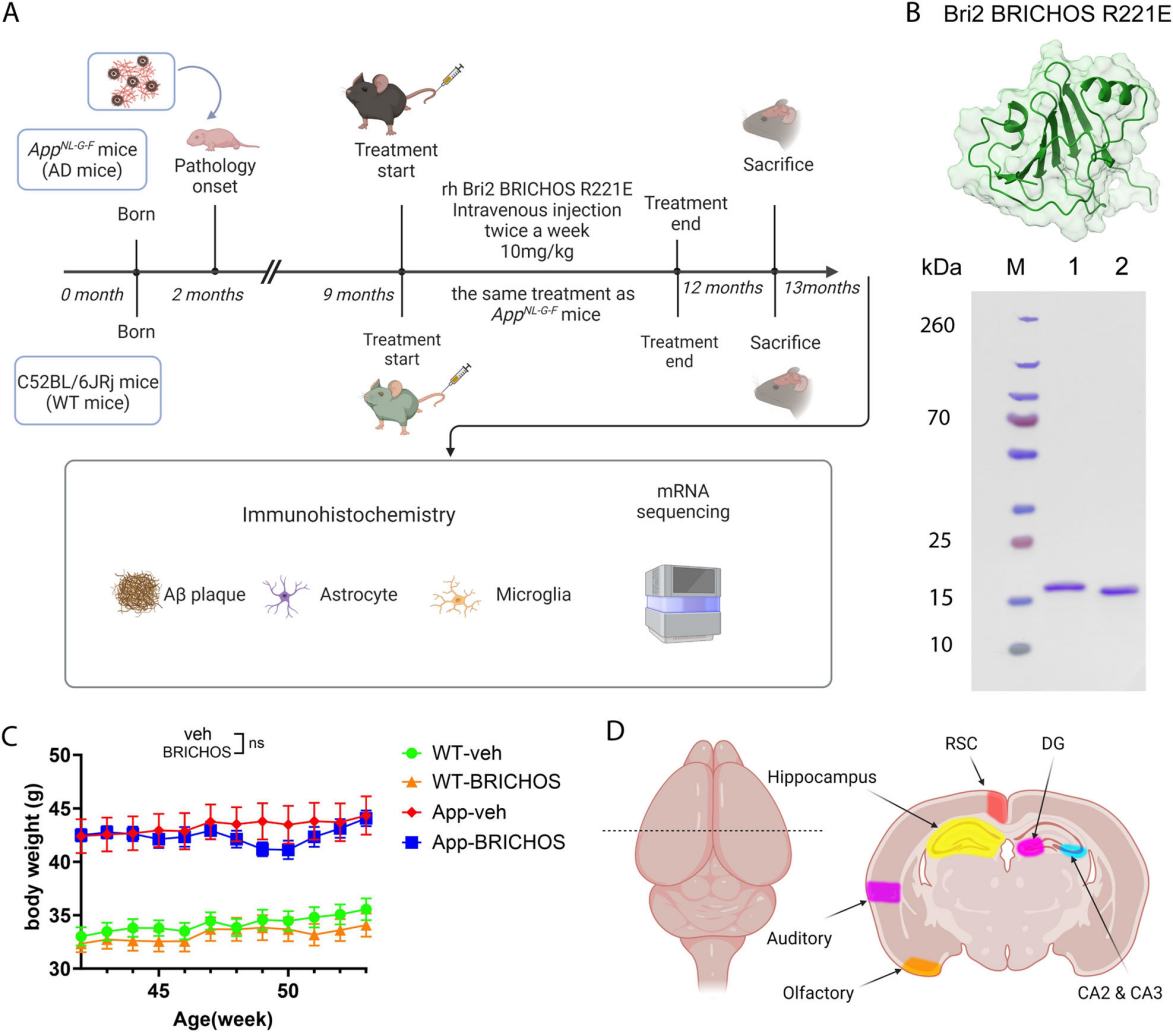
Overview of rh Bri2 BRICHOS R221E treatment of old *App*^*NL-G-F*^ and WT mice. **A** Timeline of treatment with rh Bri2 BRICHOS R221E monomer or administration of vehicle (phosphate-buffered saline, PBS) in *App*^*NL-G-F*^ and age-matched WT mice. **B** Rh Bri2 BRICHOS R221E monomer analysis by reducing (lane 1) and non-reducing (lane 2) SDS-PAGE. M shows the migration of marker proteins with molecular masses in kDa given to the left. The Bri2 BRICHOS R221E structure at the top was predicted by Alphafold2 [93]. **C** Body weight of the vehicle (veh)- or rh Bri2 BRICHOS R221E-treated *App*^*NL-G-F*^ (App) and WT mice during the treatment period, presented as mean ± SEM. Two-way ANOVA with Tukey’s correction for multiple comparisons was used to calculate *p*-values for differences in body weight. ns no significance. **D** Areas of the hippocampus and cerebral cortex analysed by immunohistochemistry. Auditory auditory cortex, CA *Cornu ammunis*, DG dentate gyrus, Olfactory olfactory cortex, RSC retrosplenial cortex.

The aim of the current study was to assess the ability of rh Bri2 BRICHOS R221E to affect pathology in an AD mouse model at a stage when pathology is well established. An *App* knock-in mouse model harbouring the Swedish, Iberian, and Arctic mutations (*App*^*NL-G-F*^ mouse model), which develops AD-like pathology from the second month of age, and reaches near-maximum levels by the seventh month of age [28] was used. Nine-month-old *App*^*NL-G-F*^ mice were treated i.v. with rh Bri2 BRICHOS R221E monomers, or saline, for three months and then sacrificed at thirteen months of age, i.e. one month after the last dose. Age-matched wild-type (WT) mice were treated in the same way. Analysis of amyloid plaque load, reactive gliosis, and bulk microglia RNA sequencing (Fig. 1A), indicate that rh Bri2 BRICHOS treatment is efficient at a late AD-like stage and works by targeting Aβ-induced pathology.

## Results

### Intravenous BRICHOS R221E administration to aged *App*^*NL-G-F*^ mice

The *App*^*NL-G-F*^ model produces Aβ toxicity by augmenting the overall production of human Aβ, shifting the Aβ_42_/Aβ_40_ ratio in favour of the more toxic Aβ_42_ form, and increasing Aβ_42_ aggregation propensity [29]. Consequently, robust amyloidosis and pathological changes start to develop at two months of age and reach near-maximal levels by the seventh month [28]. In order to assess the therapeutic potential of rh Bri2 BRICHOS R221E, we started treatment of *App*^*NL-G-F*^ mice at 9 months, and WT mice were treated in the same way, to enable investigation of potential effects not related to the AD-like pathology (Fig. 1A).

Rh Bri2 BRICHOS R221E was produced in *E. coli* and subsequently purified using immobilized metal ion affinity chromatography and size exclusion chromatography (SEC), as previously established [27]. SDS-PAGE of the SEC-isolated rh Bri2 BRICHOS R221E monomers under reducing or non-reducing conditions, demonstrated high purity (Fig. 1B). Notably, the slightly accelerated migration observed by SDS-PAGE under non-reducing conditions indicates the presence of a conserved intramolecular disulphide bond [27]. *App*^*NL-G-F*^ and WT mice from nine months of age received i.v. administration (in the tail vein) of rh Bri2 BRICHOS R221E monomers at a dose of 10 mg/kg twice a week for a duration of twelve weeks, or vehicle (Veh, i.e. PBS) (Fig. 1A), i.e. the same dosing regimen as previously used in early-stage *App*^*NL-G-F*^ mice [24]. Throughout the treatment period, we monitored the body weight of the mice once a week. Generally, both the AD model mice and their WT counterparts tolerated the rh Bri2 BRICHOS R221E injections. Rh Bri2 BRICHOS-treated *App*^*NL-G-F*^ mice displayed a 5–10% decrease in body weight after 8–9 weeks of treatment compared to those given PBS (*p*-values 0.5 and 0.6, respectively), followed by a subsequent recovery (Fig. 1C). One month after the completion of the treatment regimen, the mice were sacrificed and immunohistochemical, biochemical and transcriptome analyses on various regions of the hippocampus and cortex, were conducted, i.e. the same interval was employed between sacrifice and analyses as previously used in early-stage *App*^*NL-G-F*^ and late stage *App*^*NL-F*^ mice [24]. The regions analysed included the dentate gyrus (DG), *Cornu ammonis* (CA) regions 2 and 3 (CA2 and CA3) of the hippocampus, the retrosplenial cortex (RSC), and the auditory and olfactory cortex areas (Fig. 1D).

### Rh Bri2 BRICHOS R221E passes the BBB and reduces Aβ plaque load

**Fig. 2:**
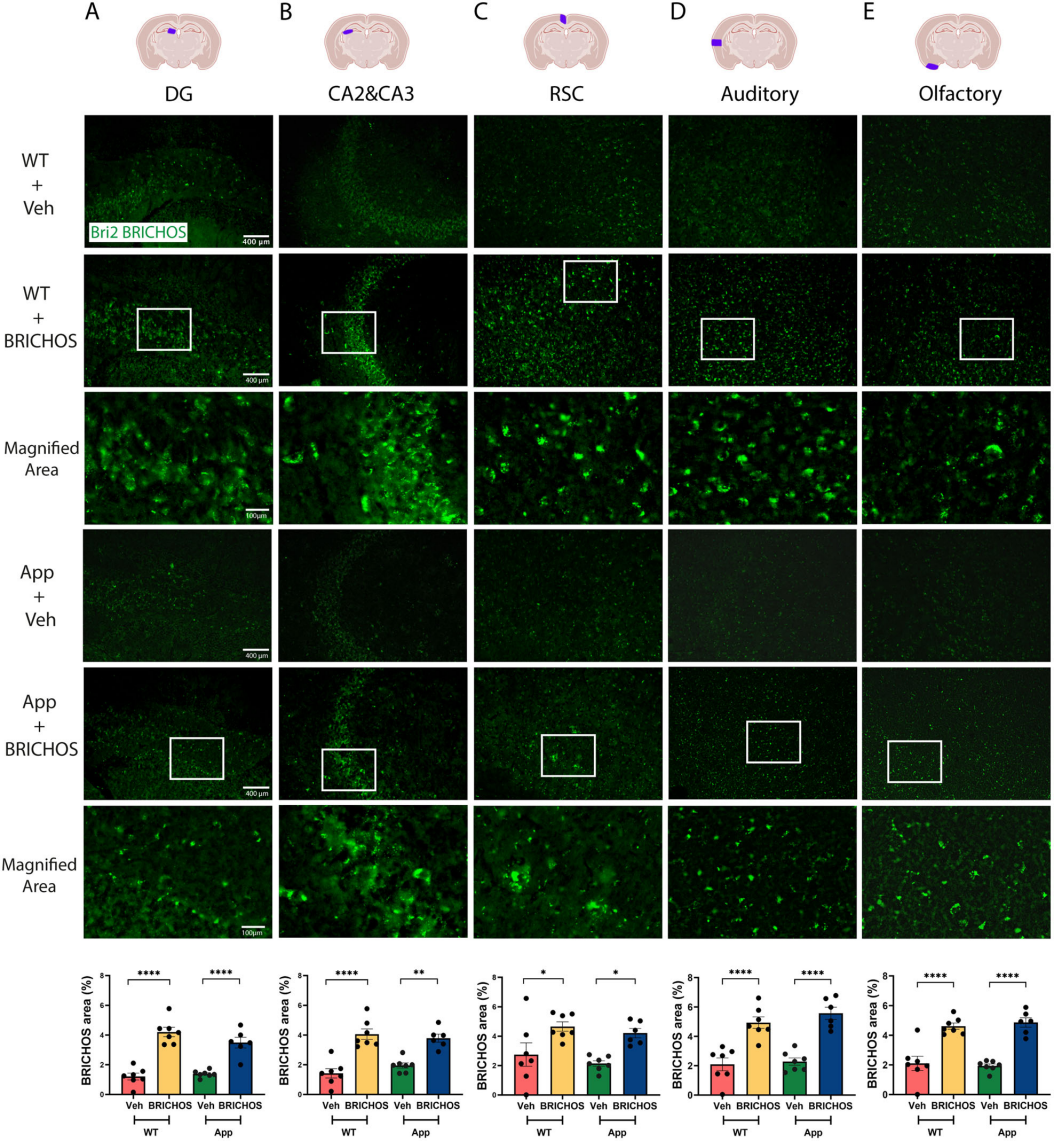
Rh Bri2 BRICHOS R221E accumulates in the brains of *App*^*NL-G-F*^ and WT mice after intravenous injections. Representative images of sections from indicated brain regions (column **A**, DG and **B**, CA2 and CA3 hippocampal regions; **C**, RSC, **D**, auditory and **E**, olfactory cortex regions) of rh Bri2 BRICHOS R221E- or vehicle (veh)-treated *App*^*NL-G-F*^ (App) and WT mice stained with anti-Bri2 BRICHOS antibody. The areas marked with boxes are shown in the panels below them. The punctuate pattern of staining indicates that rh Bri2 BRICHOS R221E is present intracellularly, see text for details. The bar charts show the rh Bri2 BRICHOS-positive area (% of total area) in the different brain regions for each treatment group. Scale bars represent 400 μm and apply to all panels in the respective column, except the magnified areas for which the scale bars are 100 μm. Data are shown as mean ± SEM (*n* = 6–7 mice per group). One-way ANOVA test and Tukey’s test were used to calculate the *p*-values. ^∗^*p* < 0.05, ^∗∗^*p* < 0.01, ^∗∗∗∗^*p* < 0.0001. Auditory auditory cortex, CA *Cornu ammunis*, DG dentate gyrus, Olfactory olfactory cortex, RSC retrosplenial cortex.

To assess the BBB permeability of rh Bri2 BRICHOS R221E following i.v. administration in both WT and *App*^*NL-G-F*^ mice, brain sections were subjected to immunohistochemistry for Bri2 BRICHOS and subsequent quantification. The results revealed higher levels of Bri2 BRICHOS staining in the cortex and in DG, CA2, and CA3 regions of the hippocampus of *App*^*NL-G-F*^ and WT mice after rh Bri2 BRICHOS R221E treatment compared to mice receiving PBS (Fig. 2A–E). Notably, no differences in Bri2 BRICHOS staining were observed between the WT and *App*^*NL-G-F*^ mice, suggesting that the BBB of *App*^*NL-G-F*^ mice has a similar permeability as that of WT mice. These findings strongly suggest that i.v. administered rh Bri2 BRICHOS R221E effectively crosses the BBB and is retained in the brain for at least one month in both WT and *App*^*NL-G-F*^ mice. The same degree of increase in Bri2 BRICHOS staining in *App*^*NL-G-F*^ and WT mice (Fig. 2) was observed, in spite of the fact that rh Bri2 BRICHOS R221E binds to Aβ_42_ fibrils [30, 31]. This is likely related to that i.v. injected rh Bri2 BRICHOS is internalized into neurons [32] and only a small fraction (ca.10%) of added rh Bri2 BRICHOS R221E binds to Aβ_42_ fibrils [31].

**Fig. 3:**
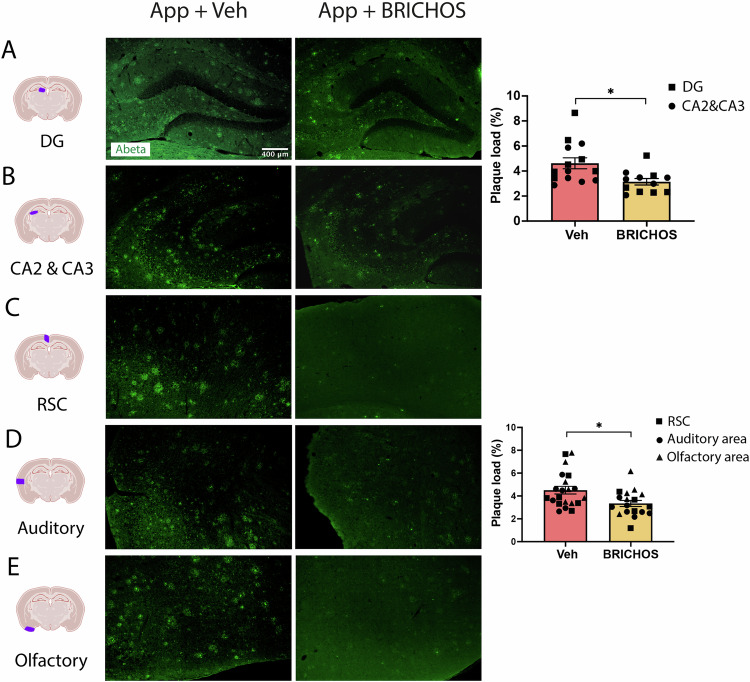
Plaque load is reduced in *App*^*NL-G-F*^ mice after rh Bri2 BRICHOS R221E treatment. Representative images of the hippocampus (**A**, **B**) and cerebral cortex (**C**–**E**) sections from vehicle (veh)- and rh Bri2 BRICHOS R221E-treated *App*^*NL-G-F*^ (App) mice stained with anti-Aβ antibody 82E1. Histograms show plaque load (%) in different hippocampal and cortical regions. The scale bar represents 400 μm and applies to all panels in (**A**–**E**). Data are presented as mean ± SEM (*n* = 6–7 mice/group). An unpaired parametric two-tailed *t*-test was used for statistical analysis. ^∗^*p* < 0.05. Auditory auditory cortex, CA *Cornu ammunis*, DG dentate gyrus, Olfactory olfactory cortex, RSC retrosplenial cortex.

Rh Bri2 BRICHOS R221E has been shown to delay Aβ_42_ amyloid fibril formation in vitro [15], and administration to *App*^*NL-G-F*^ mice starting at three months of age reduced amyloid plaque burden [24]. To investigate the effects of rh Bri2 BRICHOS R221E in older *App*^*NL-G-F*^ mice, brain sections were stained with the Aβ antibody 82E1. The results demonstrated a reduced load of Aβ plaques in the DG, CA2 and CA3 regions of the hippocampus (Fig. 3A, B) and cortex (both auditory and olfactory) (Fig. 3C–E) of mice treated with rh Bri2 BRICHOS R221E compared to PBS. The degree of reduction in the treatment of older *App*^*NL-G-F*^ mice (Fig. 3) is comparable to the effect sizes in early-stage *App*^*NL-G-F*^ mice [24]. These results suggest that the i.v. administration of rh Bri2 BRICHOS R221E reduces amyloid burden in aged *App*^*NL-G-F*^ mice also when amyloid pathology had nearly reached saturation [28] at the start of treatment.

### Rh Bri2 BRICHOS R221E treatment of aged *App*^*NL-G-F*^ mice reduces microgliosis and astrogliosis

**Fig. 4:**
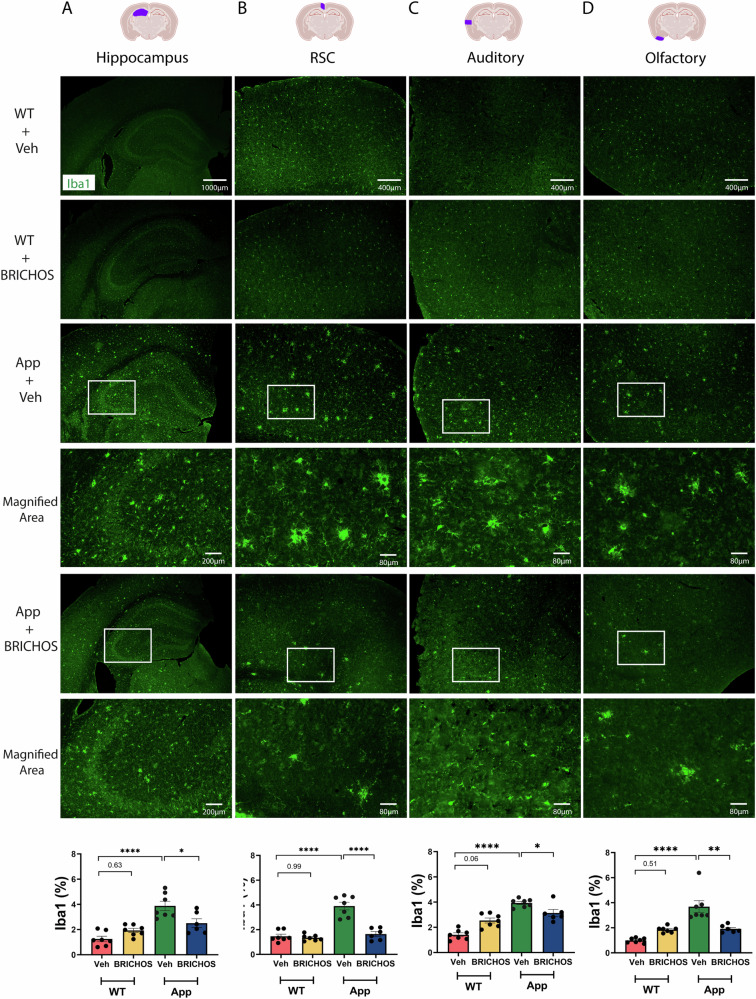
Rh Bri2 BRICHOS R221E treatment mitigates microgliosis in *App*^*NL-G-F*^ mice. Representative images of hippocampus (**A**) and cortex (**B**–**D**) sections from vehicle (veh)- and rh Bri2 BRICHOS R221E-treated *App*^*NL-G-F*^ (App) and WT mice stained by immunohistochemistry for Iba1. Bar charts at the bottom show the Iba1-positive area (%) in each brain region for the four treatment groups. The areas marked with boxes are magnified in the panels shown below them. All micrographs from the hippocampus are at a lower magnification than the micrographs from the cortex. Scale bars represent 1000 μm (**A**) and 400 μm (**B**–**D**) and apply to all panels in the respective column, except the magnified areas for which scale bars represent 200 μm (**A**) or 80 μm (**B**–**D**). Data are shown as mean ± SEM (*n* = 6–7 mice/group). One-way ANOVA test and Tukey’s test were used for statistical analysis. ^∗^*p* < 0.05, ^∗∗^*p* < 0.01, ^∗∗∗∗^*p* < 0.0001. The entire hippocampus was analysed. Iba1 ionized calcium-binding adaptor molecule 1, RSC retrosplenial cortex.

To further evaluate the therapeutic effects of rh Bri2 BRICHOS R221E in aged *App*^*NL-G-F*^ mice, we analysed the degree of microgliosis by immunohistochemistry for the ionized calcium-binding adaptor molecule 1 (Iba1), a marker for microglial activation. This clearly showed an overall reduction in Iba1-positive microglia in both the hippocampus and cortex areas after rh Bri2 BRICHOS R221E treatment, compared to the PBS controls, while rh Bri2 BRICHOS R221E treatment of WT mice did not change the levels of Iba1 immunoreactivity (Fig. 4).

**Fig. 5:**
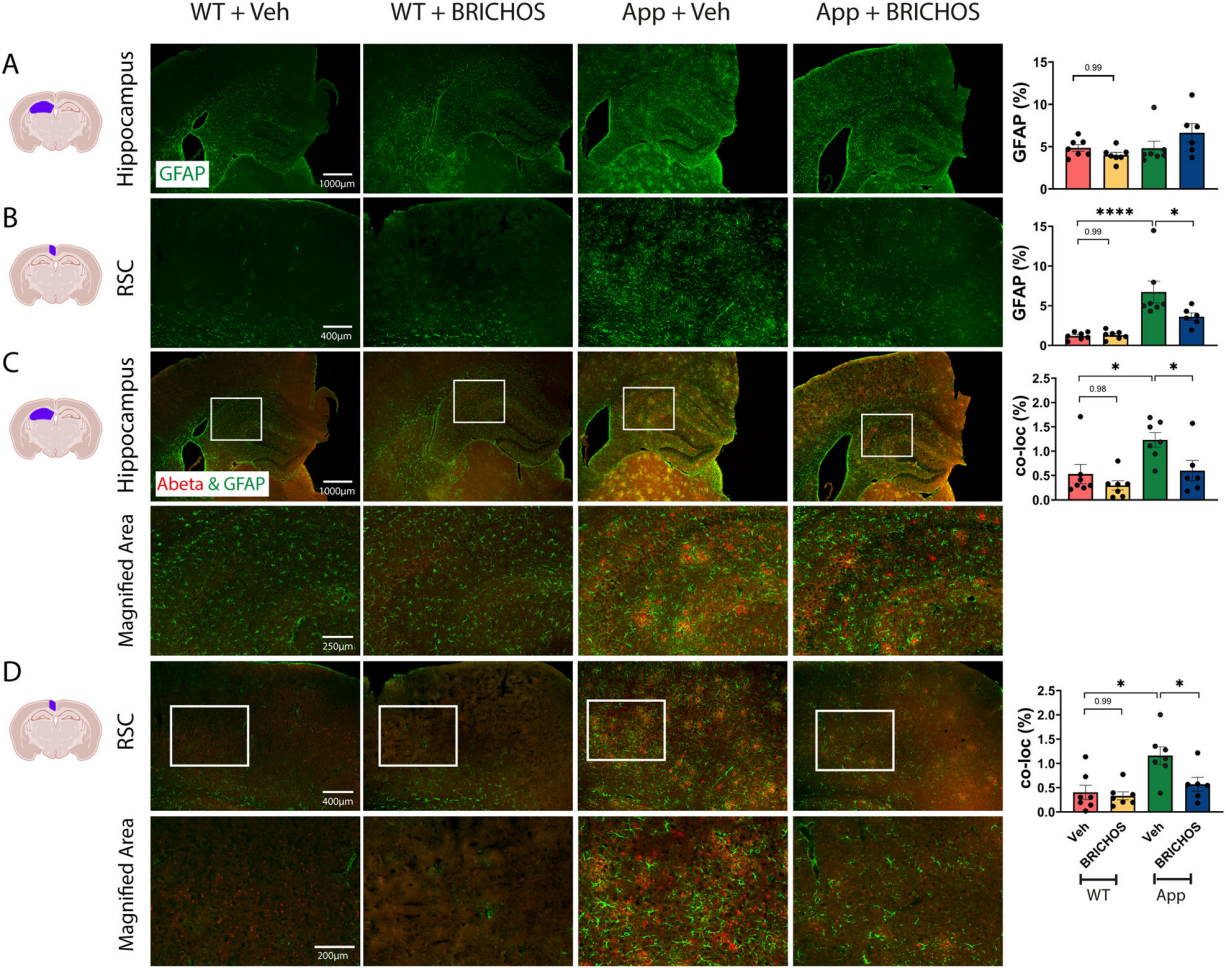
Rh Bri2 BRICHOS R221E treatment mitigates astrogliosis in *App*^*NL-G-F*^ mice. Representative images of brain sections from vehicle (veh)- and rh Bri2 BRICHOS R221E-treated *App*^*NL-G-F*^ (App) and WT mice stained by immunohistochemistry for GFAP (green, rows **A**, **B**) and for GFAP and Aβ (green and red, rows **C**, **D**). The areas marked with boxes in (**C**, **D**) are magnified in the panels shown below them. Scale bars represent 1000 μm (**A**, **C**), 400 μm (**B**, **D**), and 200 μm (magnified areas) and apply to all panels in the respective row. Bar charts to the right show the GFAP-positive area (%) in the hippocampus (**A**) and retrosplenial cortex (RSC) (**B**), or the GFAP and Aβ colocalized (co-loc) area in the hippocampus (**C**) and RSC (**D**). Data are shown as mean ± SEM (*n* = 6–7 mice/group). One-way ANOVA test and Tukey’s test were used for statistical analysis. ^∗^*p* < 0.05, ^∗∗∗∗^*p* < 0.0001. The entire hippocampus was analysed. GFAP glial fibrillary acidic protein, RSC retrosplenial cortex.

Next, we analysed the degree of astrogliosis using the astrocyte marker glial fibrillary acidic protein (GFAP). In *App*^*NL-G-F*^ mice treated with rh Bri2 BRICHOS R221E, the levels of GFAP immunoreactivity decreased in the RSC area, while there was no significant change in the entire hippocampus (analysed as whole area) (Fig. 5A, B), nor in the auditory or olfactory cortex areas (not shown) compared to the PBS control group. In order to explore more specifically the effects on astrocytes in the vicinity of Aβ plaques, we double-stained brain sections for GFAP and Aβ and analysed the degree of colocalization of immunoreactivity. The results showed increased colocalization of GFAP and Aβ staining in PBS-treated *App*^*NL-G-F*^ mice compared to WT mice, and significantly less colocalization of Aβ and GFAP staining in the entire hippocampus and RSC of *App*^*NL-G-F*^ mice after rh Bri2 BRICHOS R221E treatment (Fig. 5C, D). Similar to Iba1, GFAP immunoreactivity in WT mice was not affected by rh Bri2 BRICHOS R221E treatment (Fig. 5).

Taken together, these results indicate that i.v. rh Bri2 BRICHOS R221E treatment was able to reduce Aβ-induced astrogliosis and microgliosis in aged *App*^*NL-G-F*^ mice.

### Impact of rh Bri2 BRICHOS R221E treatment on gene expression in microglia differs between WT and *App*^*NL-G-F*^ mice

**Fig. 6:**
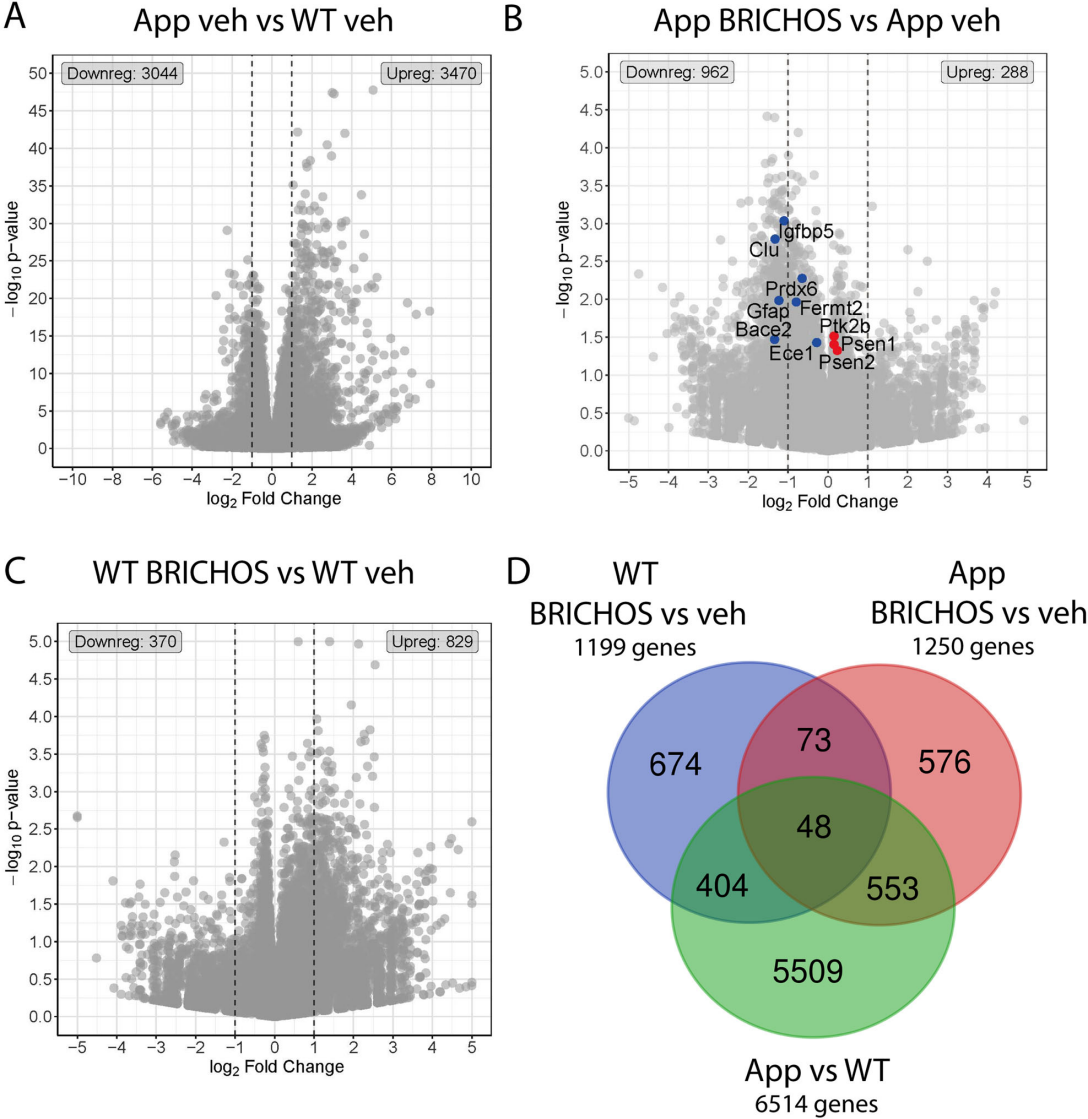
Differentially expressed genes (DEGs) in microglia from *App*^*NL-G-F*^ and WT mice. **A**–**C** Volcano plots showing DEGs between **A** vehicle (veh)-treated *App*^*NL-G-F*^ (App) and WT mice; **B** rh Bri2 BRICHOS R221E- and veh-treated *App*^*NL-G-F*^ mice, and **C** rh Bri2 BRICHOS R221E- and veh-treated WT mice. *N* = 3 mice/group. Different scales are used in panels (**A**–**C**) for optimal resolution of gene symbols. Vertical dotted lines indicate | log_2_fold change | ≥1 border. The ten genes identified in panel (**B**) are further analysed in Fig. 8. The number of down- or up-regulated genes is indicated for each panel. **D** Venn diagrams of DEGs in veh-treated WT and *App*^*NL-G-F*^ mice (green circle), veh- and rh Bri2 BRICHOS R221E-treated *App*^*NL-G-F*^ mice (red circle), and veh- and rh Bri2 BRICHOS R221E-treated WT mice (blue circle). The identities of the genes in the different intersections between the circles are given in the Supplementary Excel file 1.

As we observed robust effects of rh Bri2 BRICHOS R221E treatment on microglial activation (Fig. 4), we isolated cortical microglial cells from three mice from each of the four treatment groups, i.e. WT and *App*^*NL-G-F*^ mice treated with either PBS (veh) or rh Bri2 BRICHOS R221E, and performed bulk mRNA sequencing. Comparison of *App*^*NL-G-F*^ with age-matched WT mice, both receiving veh, using a *p*-value threshold of < 0.05, identified 3470 upregulated genes and 3044 downregulated genes (Fig. 6A). Using the same *p*-value cut-off we found 288 upregulated genes and 962 downregulated genes between treatment with rh Bri2 BRICHOS R221E or veh in *App*^*NL-G-F*^ mice, and 829 upregulated genes and 370 downregulated genes between treatment with rh Bri2 BRICHOS R221E or veh in WT mice (Fig. 6B, C). There is a 5–7-fold higher number of genes affected in *App*^*NL-G-F*^ vs WT mice compared to the number of genes affected by rh Bri2 BRICHOS R221E vs PBS treatment of *App*^*NL-G-F*^ or WT mice (Fig. 6A–C). This is likely explained by the more profound (genetic) differences between *App*^*NL-G-F*^ and WT mice, compared to the three months of rh Bri2 BRICHOS R221E or PBS treatment. Cluster heatmap analysis of the 100 genes that showed the highest variance between all four treatment groups (Supplementary Fig. 1) confirmed that mouse strain (*App*^*NL-G-F*^ vs WT) had a stronger influence on gene expression levels as compared to treatment (rh Bri2 BRICHOS R221E vs veh). Microglial genes associated with AD, including *Trem2*, *Spp1*, *Tyrobp*, and *Cd74*, were among the upregulated genes in *App*^*NL-G-F*^ vs WT mice, supporting the activation of microglia in *App*^*NL-G-F*^ mice, consistent with previous findings [33–35].

The 1250 differentially expressed genes (DEGs) in *App*^*NL-G-F*^ mice treated with rh Bri2 BRICHOS R221E were mainly downregulated, while the same treatment of WT mice resulted in 1199 DEGs, and most of them were upregulated (Fig. 6B–D). This indicates that rh Bri2 BRICHOS R221E treatment affects AD-relevant genes that are upregulated in the *App*^*NL-G-F*^ mice vs WT mice. The 48/121 DEGs that overlapped between rh Bri2 BRICHOS R221E vs veh treated WT mice and rh Bri2 BRICHOS R221E vs veh treated *App*^*NL-G-F*^ mice and also are differentially regulated in *App*^*NL-G-F*^ vs WT mice (Fig. 6D) represent several different biological processes without known relevance for AD-like pathology (Supplementary Excel file 1). Finally, gene set enrichment analysis [36, 37] of the DEGs between treatment with rh Bri2 BRICHOS R221E or veh in WT mice (Supplementary Fig. 2) showed that many of them were associated with cellular shape and movement processes and membrane channel functions.

### Rh Bri2 BRICHOS R221E treatment of *App*^*NL-G-F*^ mice affects the expression of AD-related genes in microglia

**Fig. 7:**
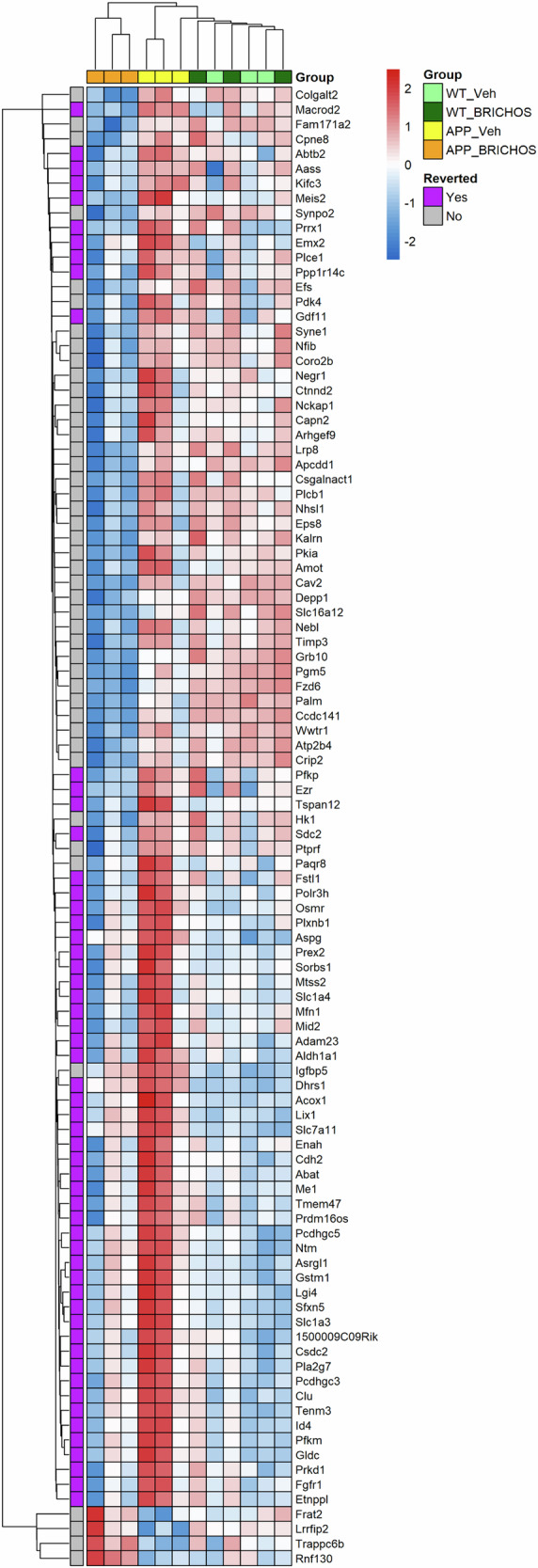
Effects of Rh Bri2 BRICHOS R221E treatment on gene expression. Heatmap for all four treatment groups for the 100 top differentially expressed genes (DEGs) between rh Bri2 BRICHOS R221E- and vehicle (veh)-treated *App*^*NL-G-F*^ (App) and WT mice. The genes were selected based on *p*-values for changed gene expression between *App*^*NL-G-F*^ mice given veh or BRICHOS, with the gene order determined by the clustering outcome. The genes reverted by rh Bri2 BRICHOS R221E treatment are further analysed in Supplementary Fig. 3 and discussed in the text. A row-based pseudo-scale has been used for the heatmap colouring, with the highest expression in red and the lowest in blue. *n* = 3 mice/group. Heat colouring is shown as | log2fold change | ≥1 and *p* < 0.05.

To further analyse the DEGs in *App*^*NL-G-F*^ mice as a result of rh Bri2 BRICHOS R221E treatment compared to veh (Fig. 6B, D), we performed a cluster heatmap analysis of the 100 most altered of these genes (Fig. 7). These 100 genes were selected based on the lowest *p*-values of fold changed expression between *App*^*NL-G-F*^ rh Bri2 BRICHOS R221E and *App*^*NL-G-F*^ veh groups. Inspection of the heatmap in Fig. 7 shows that many of the DEGs in *App*^*NL-G-F*^ mice as a result of rh Bri2 BRICHOS R221E treatment appear to change their expression levels towards normalization, i.e. they approach the levels observed in WT mice treated with either rh Bri2 BRICHOS R221E or veh. Indeed, 57 of the 100 DEGs in Fig. 7 (marked as reverted) fulfilled the criteria that they showed (i) increased expression in veh-treated *App*^*NL-G-F*^ mice compared to veh-treated WT mice, and (ii) altered expression in rh Bri2 BRICHOS R221E-treated *App*^*NL-G-F*^ mice so that the expression levels approached that in veh-treated WT mice. Bar charts of the expression levels of these 57 genes in the four different treatment groups (Supplementary Fig. 3) show that in all cases the expression levels after rh Bri2 BRICHOS R221E treatment of *App*^*NL-G-F*^ mice were fully reverted to the levels in the veh-treated WT mice. Moreover, for essentially all of these 57 genes the expression levels were the same for rh Bri2 BRICHOS R221E- and veh-treated WT mice, corroborating the conclusion from analyses of the DEGs shown in Fig. 6 that rh Bri2 BRICHOS R221E treatment largely affects DEG´s between *App*^*NL-G-F*^ and WT mice. Next, we searched references to these 57 genes within the existing literature on genes relevant for AD or for Aβ aggregation or toxicity. This revealed that 20 of the 57 genes had been concluded to be relevant for the pathogenesis of AD and/or for Aβ aggregation or toxicity (Supplementary Table 1).

### Comparison of genes affected by rh Bri2 BRICHOS R221E treatment with plaque-induced genes and genes specific for subpopulations of microglia

**Fig. 8:**
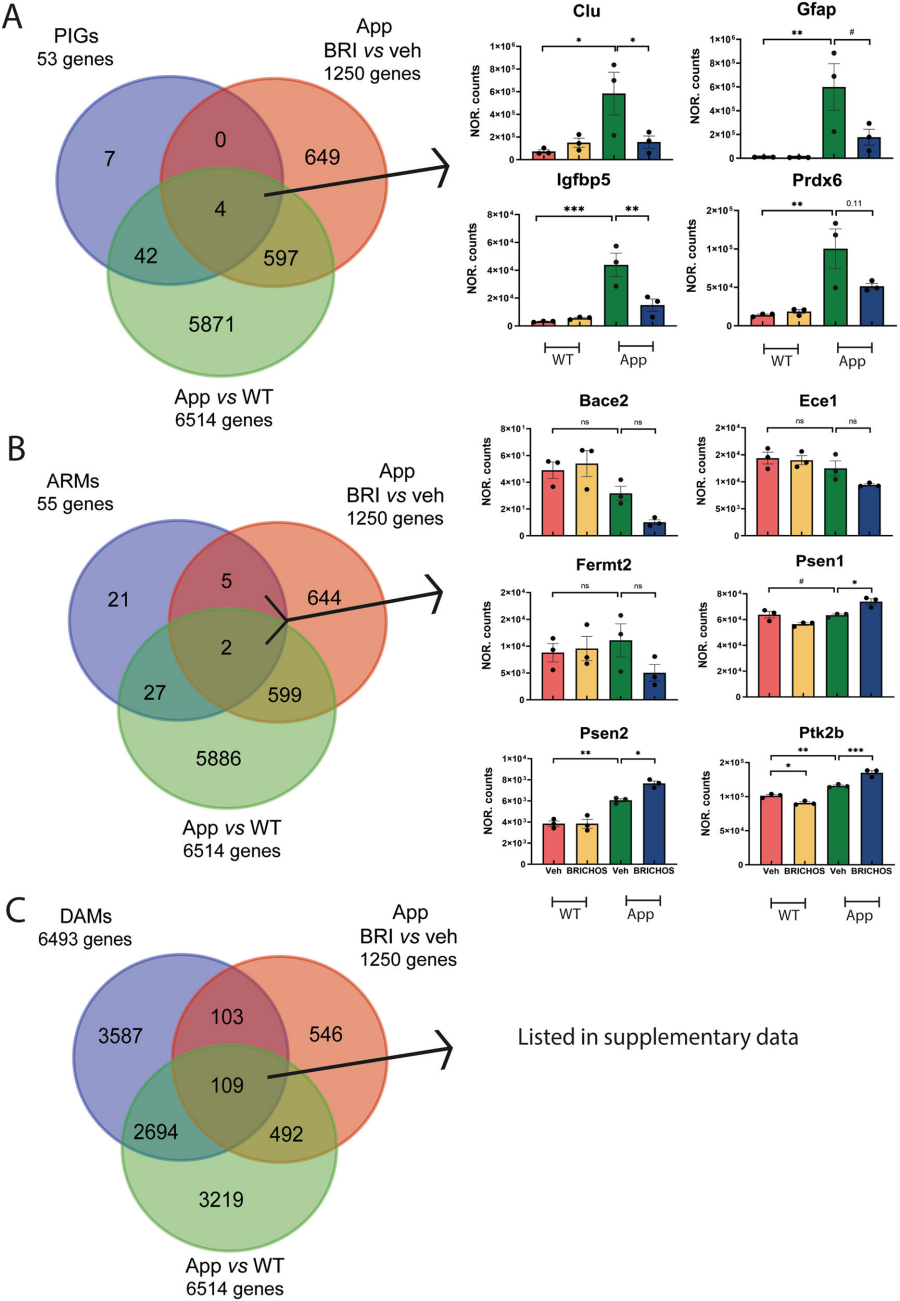
Genes affected by rh Bri BRICHOS R221E treatment in relation to plaque-induced genes and genes in microglia subpopulations. Venn diagrams illustrating differentially expressed genes (DEGs) in the vehicle (veh)-treated WT and *App*^*NL-G-F*^ (App) mice (green circle), veh- and rh Bri2 BRICHOS R221E-treated *App*^*NL-G-F*^ mice (red circle), and **A** plaque-induced genes (PIGs, blue circle); **B** activated response microglia genes (ARMs, blue circle); and **C** disease-associated microglia genes (DAMs, blue circle). Histograms of normalized (NOR) counts for the four treatment groups of genes in the indicated intersections for panels (**A**, **B**) are shown to the right. For panel **C**, the genes in the indicated intersection are identified in Supplementary Excel file 2, together with genes in intersections from panels (**A**, **B**). See main text for details. *n* = 3 mice/group, one-way ANOVA test and Tukey’s test were used for statistical analysis. ^∗^*p* < 0.05, ^∗∗^*p* < 0.01, ^∗∗∗^*p* < 0.001, 0.05 < ^#^*p* < 0.1.

Next, we compared the 1250 microglial DEGs identified between treatment with rh Bri2 BRICHOS R221E or veh in *App*^*NL-G-F*^ mice (Fig. 6B, D) with those identified in previously published studies of genes and microglial populations found to be relevant in AD and AD model mice (Fig. 8). Chen et al. [33] identified so-called plaque-induced genes (PIGs) that were found to be relevant both for *App*^*NL-G-F*^ mice and human AD. Four of the 53 PIGs [33] were found among the 1250 DEGs in *App*^*NL-G-F*^ mice treated with rh Bri2 BRICHOS R221E vs veh as well as in the *App*^*NL-G-F*^ vs WT mice treated with veh (Fig. 8A). Bar charts of the expression levels of these four genes (*Clu*, *Gfap*, *Igfbp5*, and *Prdx6*) in the four different treatment groups (Fig. 8A) show that they are upregulated in *App*^*NL-G-F*^ vs WT mice, and, except for *Prdx6*, significantly downregulated by treatment with rh Bri2 BRICHOS R221E towards the levels seen in veh-treated WT mice, while the treatment of WT mice caused no change in the expression levels.

The expression patterns of the four PIGs affected by rh Bri2 BRICHOS R221E in Fig. 8A may indicate that rh Bri2 BRICHOS treatment affects the plaques so that the release of compounds that can activate microglia is reduced, which would be in accordance with the ability of rh Bri2 BRICHOS R221E to block fibril surface catalysed generation of toxic Aβ_42_ species [38, 39]. The expression levels of the PIGs *Trem2* and *ApoE*, both known to be associated with risk of developing AD, were increased in *App*^*NL-G-F*^ mice compared to WT mice, and treatment with rh Bri2 BRICHOS R221E resulted in a further increase, ca. 20% (Supplementary Fig. 4). The effects of rh Bri2 BRICHOS R221E on *Trem2* and *Apoe*, as on *Clu, Gfap, Igfbp5* and *Prdx6* are likely related to the AD-like pathology in the *App*^*NL-G-F*^ mice, since rh Bri2 BRICHOS R221E treatment of WT mice caused no changes in *Clu, Gfap, Igfbp5* and *Prdx6*, *Trem2* or *Apoe* expression levels (Fig. 8A and Supplementary Fig. 4). Analysis of rh Bri2 BRICHOS R221E treatment effects on ligand-receptor pathways [40] (Supplementary Fig. 5) showed that receptor-mediated endocytosis and innate immunity pathways were affected in *App*^*NL-G-F*^ mice, while in WT mice the effects were linked to several different processes. Taken together, these results suggest that rh Bri2 BRICHOS R221E treatment may enhance the microglial response to amyloid deposits, promoting a more effective clearance, while at the same time mitigating overall microglial activation as seen by reduced staining for Iba1 (Fig. 4).

Activated response microglia (ARMs) [35] have been defined as a subpopulation that expresses known AD risk genes. Of the 1250 DEGs in *App*^*NL-G-F*^ mice treated with rh Bri2 BRICHOS vs veh, seven overlapped with ARMs (Fig. 8B). *Clu* is found among these genes, but three of the remaining six genes were not up- or down-regulated in *App*^*NL-G-F*^ vs WT mice, and none of them was normalized in *App*^*NL-G-F*^ mice after treatment with rh Bri2 BRICHOS R221E (Fig. 8B).

Finally, disease-associated microglia (DAMs) represent a population that can restrict neurodegeneration in AD and AD model mice [41]. Of the 1250 DEGs in *App*^*NL-G-F*^ mice treated with rh Bri2 BRICHOS vs vehicle, 212 (17%) overlap with DAMs (Fig. 8C and Supplementary Excel file 2). *Clu*, but none of the other PIGs affected by rh Bri2 BRICHOS R221E are found among these 212 genes (Supplementary Excel file 2).

## Discussion

Early diagnosis of AD presents a challenge due to the subtle nature of symptoms, often initially attributed to normal ageing. Consequently, AD is typically identified in its later stages, when individuals begin to undergo noticeable cognitive decline and memory impairment [42]. In the current study, we explored intravenous administration of rh Bri2 BRICHOS R221E in a late-stage AD mouse model. The results revealed improvements across various AD-related parameters, encompassing reduction in Aβ plaque deposition, and mitigated overall astro- and microgliosis, as well as modulation of AD-relevant microglial gene expression. The BRICHOS domain, in particular rh Bri2 BRICHOS R221E, thus emerges as a compelling candidate for the treatment of AD, holding promises also against the challenges posed by late-stage pathology.

The recent positive treatment effects in phase III clinical trials using monoclonal antibodies [2] suggest that the approach to target Aβ represents a valid strategy for treating AD, particularly in its early stages. The BRICHOS domain has been demonstrated to inhibit Aβ from forming amyloid fibrils by binding to the fibril surface, affecting secondary nucleation, a major pathway for generating neurotoxic amyloid oligomers [27, 30, 31, 43]. Reactive astrocytes and microglia are frequently observed in close vicinity to Aβ plaques in cases of AD [44–47]. The intrinsic pro-inflammatory properties of Aβ, coupled with its neurotoxic effects, lead to persistent activation of microglia, astrocyte proliferation, and a continuous release of inflammatory factors. This, in turn, contributes to further Aβ production [11]. Rh Bri2 BRICHOS R221E was shown to reduce plaque load and neuroinflammation, and to improve memory and cognition when starting the treatment at 3 months of age in *App*^*NL-G-F*^ mice [24]. Importantly, our current study markedly expanded the potential treatment window of rh Bri2 BRICHOS R221E to advanced AD stages.

Microglia participate in brain inflammation and are instrumental in the clearance of cellular debris [48, 49]. Genome-wide association studies (GWAS) revealed that a majority of AD risk genes are predominantly or exclusively expressed in microglia [50], suggesting that microglia play a significant role in AD development. Despite the advantageous functions of microglia, such as phagocytosis of Aβ, their excessive or unmanaged activation and proliferation can lead to the release of pro-inflammatory mediators, thereby contributing to neuronal cell death [11] and induction of Aβ production [51]. Here we show that treatment with rh Bri2 BRICHOS R221E resulted in an overall reduction in microglial activation, as evidenced by Iba1 immunohistochemistry, as well as altered expression levels of several PIGs.

The normalisation of expression of *Clu, Gfap, Igfbp5* and *Prdx6* by treatment with rh Bri2 BRICHOS R221E is noteworthy. The risk of developing AD is associated with common polymorphisms within the human clusterin gene (*CLU*) [52, 53]. Clusterin, also known as apolipoprotein J (ApoJ), can bind Aβ and affect its solubility, thereby preventing the formation of amyloid fibrils [54–56], suggesting that it is protective against amyloid-mediated neurotoxicity [57]. CLU upregulation may play a protective function [52, 58–60], but a correlation between increased CLU and brain atrophy and rapid clinical progression in AD patients [61] has also been observed. GFAP, suggested as a blood-based biomarker of AD [62], is upregulated in reactive astrocytosis, and a component of the neuropathological alterations seen in AD [63]. Plasma GFAP concentrations are elevated in individuals with AD or at risk of developing AD, and the levels apparently correlate with cognitive impairment [64–68]. Moreover, a close association between the concentration of GFAP in plasma and the decline in cognitive function in patients with AD has been observed [64, 65, 69, 70]. Further studies are needed of the gene expression patterns in other cells and brain areas than the cortical microglia now analysed. It will also be important to find out whether any of the gene expression changes detected here, in particular down-regulation of *Gfap*, is associated with altered GFAP levels in plasma or CSF. Insulin-like growth factor binding protein 5 (IGFBP-5) has been found to be upregulated in the hippocampus and cortex of an AD mouse model and in the cerebrospinal fluid of AD cases [71, 72]. This could contribute to neurodegeneration through oxidative stress, Aβ toxicity, and tauopathy [73, 74], and IGFBP-5 may influence AD pathology *via* direct interactions with Aβ [75]. Finally, peroxiredoxin-6 (PRDX6) has antioxidative effects and its expression is upregulated in AD [76]. The expression levels of *Prdx6* in mouse models were observed to correlate with the recruitment of astrocytes and microglia to Aβ plaques, the infiltration of these plaques by activated astrocytes, and the phagocytic activation of periplaque microglia [77].

In addition to *Clu*, *Gfap*, *Igfbp5*, and *Prdx6*, rh Bri2 BRICHOS R221E treatment also affected the expression of *Trem2* and *ApoE*. It is interesting to note that activation of the TREM2-ApoE pathway was found to shift microglia towards a phagocytic state with activity against neuritic amyloid plaques and apoptotic neurons [78], and that the ApoE3 isoform was more efficient in increasing *TREM2* expression and in mounting an Aβ-induced microglial response compared to the ApoE4 isoform, which is associated with increased risk of developing AD [79]. Moreover, it was recently shown that Bri2 BRICHOS and TREM2 proteins interact in transfected cells and that deletion of Bri2 (*ITM2B*) expression reduces phagocytosis similarly to a pathogenic *TREM2* variant [80]. The activities of phagocytosis clearance of the tissue and downregulating inflammation are generally considered part of the resolution of inflammation, see [81, 82]. Failure in this process leads to chronic inflammation, which is known as part of AD pathology [83, 84], and there are indeed experiments indicating that the resolution of inflammation is disturbed in AD [85–89]. The data presented herein suggest that that rh Bri2 BRICHOS R221E treatment enhances the microglial response to amyloid deposits, and at the same time mitigates overall microglial activation. The basis and implications of these observations require further studies.

## Materials and methods

### Animals

*App*^*NL-G-F*^ mice [28] and their background strain C57BL/6J (Jackson Laboratory, Bar Harbour, ME, USA) were maintained in temperature-controlled environments with a regular 12-h light/12-h dark cycle and with free access to food and water. The experiment was performed on male mice.

### Treatments

Forty male *App*^*NL-G-F*^ and forty WT mice were randomly selected and equally divided into four groups. The mice were treated for 12 weeks with i.v. injections of PBS (vehicle) or rh Bri2 BRICHOS R221E (10 mg/kg, *n* = 10/group) twice a week (total of 24 injections) starting from the age of 9 months. The mice were anaesthetized with 2% isoflurane by inhalation in a plastic induction chamber for a short period of time necessary to administer the i.v. injection.

After twelve weeks of treatment, and another four weeks without treatment as used in previous studies [24] the mice were anaesthetized and perfused intracardially with physiological saline. The brains were dissected out and divided into hemispheres. The left hemispheres were further dissected into the olfactory bulb, hippocampus, cerebral cortex, brain stem and cerebellum, frozen in dry ice and stored at −80 °C for biochemical analyses. The right hemispheres were fixed in 4% paraformaldehyde (PF) in 0.1 M phosphate buffer and stored at +4 °C for immunohistochemical analysis. The experimenters performing the analyses were blinded to the nature of the intervention group the sample originated from.

### Rh Bri2 BRICHOS R221E protein expression and purification

A gene coding for human Bri2, residues 113–231, with Arg at position 221 replaced with Glu, referred to as Bri2 BRICHOS R221E, was fused to a gene fragment coding for the NT^*^ solubility tag followed by a thrombin cleavage site, and expressed in Shuffle T7 *Escherichia coli* cells as described previously [27]. Shuffle T7-competent *E. coli* cells were cultured at +30 °C in lysogeny broth (LB) medium supplemented with 15 μg/mL kanamycin. The temperature was lowered to +20 °C after the optical density at 600 nm (OD600) reached 0.8–1.0, and after adding 0.5 mM isopropyl β-D-1-thiogalactopyranoside (IPTG) the cells were incubated overnight. The cells were collected and resuspended in 20 mM Tris-HCl (pH 8.0). The cells were lysed with a 5-min sonication on ice (2 s on, 2 s off, 65% power), followed by centrifugation at +4° (24,000×*g*) for 30 min. The resulting supernatant with target proteins was purified with an immobilized metal affinity chromatography (IMAC) column (Ni Sepharose 6 Fast Flow; GE Healthcare, UK) equilibrated with 20 mM Tris-HCl (pH 8.0). The fusion protein was eluted with 300 mM imidazole in 20 mM Tris-HCl (pH 8.0) and dialysed against 20 mM Tris-HCl (pH 8.0) overnight in a cold room. Subsequently, the NT*-Bri2 BRICHOS R221E fusion proteins were incubated with thrombin (1:600 enzyme-to-substrate weight ratio, Merck) for 72 h at +4 °C and applied onto a second IMAC column to remove the His_6_-NT^∗^ tag. The monomeric rh Bri2 BRICHOS R221E fractions were isolated using an ÄKTA system (GE Healthcare, UK) and a Superdex 75 PG column (GE Healthcare, UK). The resulting monomeric R221E proteins were dialysed against filtered and autoclaved PBS (pH 7.4), concentrated to the required concentration (~2 mg/mL), and filtered through a 0.2 μm Millex-GV filter (Merck Millipore, Ireland). Coomassie staining after SDS-PAGE was used to assess the final purity of rh monomeric Bri2 BRICHOS R221E.

### Immunohistochemistry

Following dissection, the right brain hemispheres were placed in 4% PF and kept at +4 °C overnight. Next, the tissues were soaked in 30% sucrose for 24 h and then moved to a cryoprotectant solution and stored at −20°C. The brain hemispheres were cut in the coronal plane using a Leica cryostat to obtain 20 μm thick sections, which were stored free-floating in the cryoprotectant at −20 °C until further processing. The sections were transferred into 24-well plates with 0.01 M PBS and after three washes they were incubated for 20 min with PBS containing 0.3% Triton X-100 at room temperature (RT). Following another PBS wash, the sections were blocked for 30 min in a solution containing 0.1% Triton X-100 in PBS (PBST) and 5% normal donkey or goat serum. The sections were then incubated overnight at +4 °C with primary antibodies. Normal donkey serum in the absence of primary antibodies was used as a negative control. After a PBS wash, the sections were incubated with secondary antibodies for one h at RT. After three washes in PBST, the sections were mounted with an anti-fading mounting medium (ProLong™ Glass Antifade Mountant, Catalogue No. P36984). The primary and secondary antibodies used are listed in Supplementary Tables 2 and 3.

### Image analysis

Images in four regions were analysed: hippocampus (approximately 1000–3800 µm posterior to bregma in the mouse brain), retrosplenial cortex (1000–2500 µm posterior to bregma), auditory cortex (around 2500–3800 µm posterior to bregma) and olfactory cortex hippocampal level (around 2800–4500 µm anterior to bregma) in coronal sections.

Images of the entire hippocampus were captured at 4× magnification and analysed for Iba1 and GFAP staining. The cortex regions, DG, and the CA2 and CA3 regions were captured at 10× and analysed for Bri2 and Aβ staining. A Nikon Eclipse microscope (Eclipse E800, Nikon, Japan) was used to capture the images. Three consecutive sections per animal were stained and subjected to quantitative analysis using Image J software (NIH, MD, USA). Background fluorescence values from negative controls (only stained with secondary antibodies) were subtracted from all images. The lowest threshold for detection of fluorescence signal from samples stained with primary and secondary antibodies was then manually determined for 20 images, including all treatment groups, and averaged. The area (in per cent of the total image area) that showed fluorescence above this threshold was averaged for three consecutive sections per animal. For each group, 6–7 animals were analysed.

Colocalization of GFAP and Aβ staining was quantitatively assessed using Image J software. Image adjustments for brightness, contrast and threshold were uniformly applied to sections double-stained with antibodies specific to GFAP and Aβ. These images were of identical dimensions and represented the same anatomical region. Subsequently, the GFAP-stained image was pseudo-coloured green and the Aβ-stained image red. Merging of these images resulted in a composite wherein areas of GFAP and Aβ colocalization appeared yellow. The percentage of the total area that showed yellow colour was then determined as for the single stainings described above, and plotted as per cent of colocalization.

### Protein extraction

Soluble and insoluble protein fractions were extracted from the hippocampus and cerebral cortex. Fresh-frozen brain samples were mechanically homogenised by a 3-step extraction protocol. TRIS-buffered saline (TBS) (20 mM Tris-HCl, 150 mM NaCl, pH 7.4) containing cocktails of phosphatase and protease inhibitors (Chemical Co., Stockholm, Sweden, and Thermo Fisher Scientific, Stockholm, Sweden, respectively) was used for homogenization of the tissues. The supernatant was collected as a TBS fraction after centrifugation (56,000 rpm, +4 °C, 20 min). The remaining pellet was homogenised in TBST (TBS containing 1% Triton X-100) and centrifuged (56,000 rpm, +4 °C, 20 min). The supernatant was collected as a TBST fraction. The remaining pellet was sonicated (15 s on, 15 s off, 15 s on, 15 s off, 20% power) in 80% formic acid and centrifuged (56,000 rpm, +4 °C, 20 min), and the resulting supernatant collected as a formic acid fraction.

### Microglia and RNA isolation

Papain dissociation kits (Worthington, Catalogue. No. LK003178) along with magnetic cell isolation were used to isolate CD11b-positive microglia from cerebral cortex samples [90] according to the manufacturer’s instructions. The cortical tissue was minced and incubated in papain enzyme solution at +37 °C for 40 min to digest the connective tissue. A single-cell pellet was collected by density gradient centrifugation. The cells were filtered through 70 μm cell strainers, incubated with magnetic beads conjugated with anti-CD11b antibodies and the CD11b-positive microglia were isolated. The microglial cells were suspended in RNA*later* solution (Thermo Fisher, Catalogue No. AM7020) and stored at −80 °C until used.

The RNeasy micro kit (Qiagen, Germany) was used to isolate and purify RNA from the isolated microglia. Procedures were performed according to the manufacturer’s specifications. The quality and purity of the RNA was measured photometrically.

### RNA-sequencing

Adaptor and read quality trimming were performed by Cutadapt (v3.5). Using MultiQC (v1.14) and FastQC (v0.11.8), the quality of the samples was evaluated. STAR (v2.7.9d) was used to align reads to the Ensembl GRCm38 reference genome. Gene counts were acquired by employing featureCounts (v1.5.1). For library size normalization of raw counts and sample group comparisons with Wald tests, the Bioconductor package DESeq2 (v1.36.0) was utilised. *p*-values adjusted for multiple testing (p_adj_) were estimated using the Benjamini-Hochberg method. For differential gene expression analysis throughout the study, we applied specific cutoffs: log_2_FC > 1 (indicating upregulation, FC = fold change, 2-fold), log_2_FC < −1 (indicating downregulation, 2-fold), and *p* < 0.05.

### Statistical analysis

All statistical analyses were performed in GraphPad Prism 10 (GraphPad, CA, USA) or Excel. The results were expressed as bar graphs as mean ± SEM. *p* < 0.05 was considered statistically significant, if not stated otherwise. Normal distribution was checked by the Shapiro–Wilk test. To analyse differences between treatments, Tukey’s test and one-way ANOVA were used to test for group differences. An unpaired parametric two-tailed *t*-test was used to test the difference between the two groups. Figure legends describe the type of statistical test, the definition of significance for various *p*-values, and the number of biological replicates (*n*) for each group. The DESeq2 comparisons and enrichment testing were done by R 4.3.1. The DESeq2 comparisons (Wald test statistics) are used for the selection of regulated genes (described in the Results section), volcano plots and enrichment analysis, while the bar chart *p*-values are based on ANOVA/*t*-tests of normalized counts. Enrichment analysis of functional groups was performed using the Bioconductor package fgsea together with records from the Molecular Signatures Database (MSigDB), accessible via Bioconductor package msigdbr [36, 91, 92]. Log2 fold changes from the DESeq2 comparisons were used as input data.

## Supplementary information

Supplemental Figures, Tables

Supplementary Excel file 1

Supplementary Excel file 2

## Data Availability

The RNA sequencing data are publically available under the GEO ID GSE263166. All data and materials related to this paper are available upon request.
