# Supplementary material for: Intravenous chaperone treatment of late-stage Alzheimer´s disease (AD) mouse model affects amyloid plaque load, reactive gliosis and AD-related genes
Source: Transl Psychiatry. 2024 Oct 24;14:453. doi: 10.1038/s41398-024-03161-x (PMC11502864; doi:10.1038/s41398-024-03161-x)
Supplement: Supplementary file 1 — Supplemental Figures, Tables [file 41398_2024_3161_MOESM1_ESM.docx]

**Intravenous chaperone treatment of late-stage Alzheimer´s disease (AD) mouse model affects amyloid plaque load, reactive gliosis and AD-related genes**

Ruixin Zhang, Makiko Ohshima, David Brodin, Yu Wang, Antonin Morancé, Marianne Schultzberg, Gefei Chen, Jan Johansson

**Supplementary Figures 1-5**

**Supplementary Tables 1-3**

**Supplementary Excel Files 1-2**

**
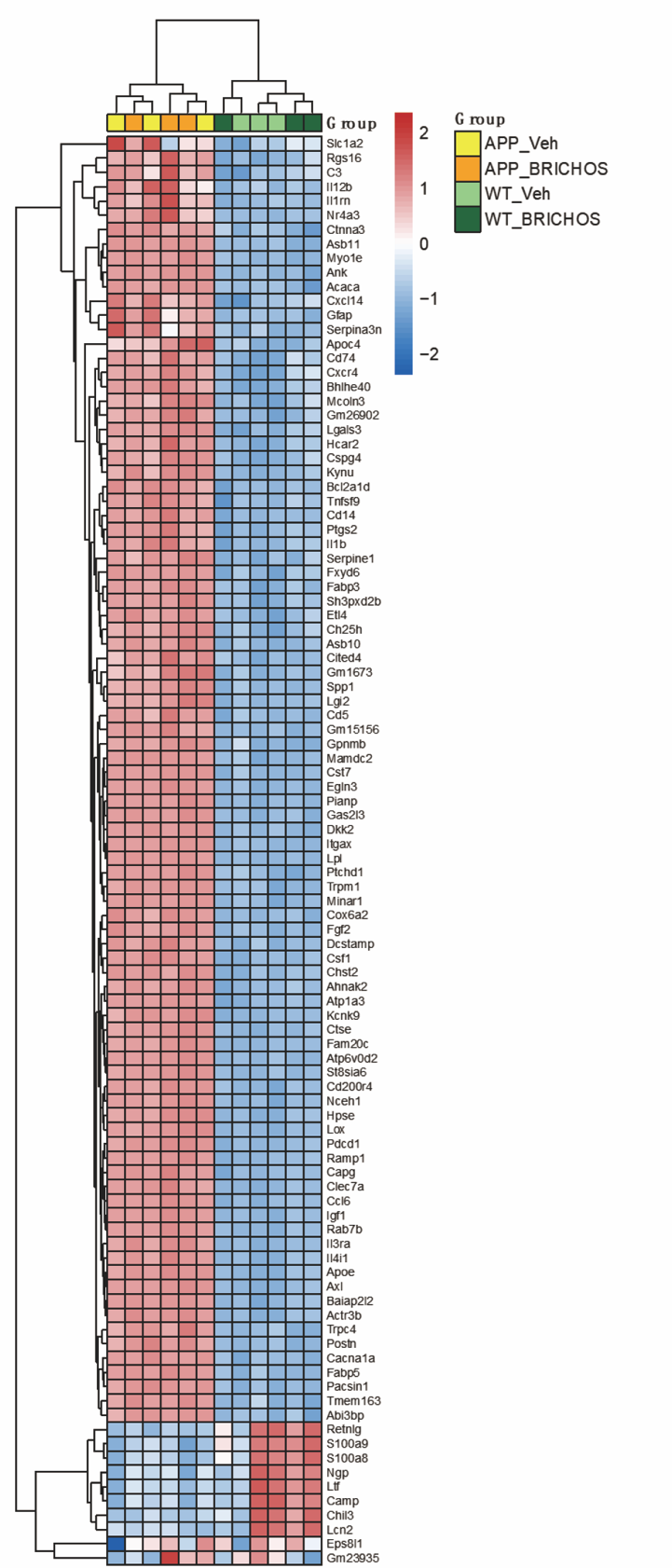
**

**Supplementary Figure 1. Largest gene expression differences between *App^NL-G-F^* and WT mice.** Heatmap with hierarchical clustering of samples and genes for the most variable genes in the four treatment groups. DESeq2's variance stabilizing transformation (VST) of count data were used for the selection of the top 100 variance genes. Heatmap coloring is based on a per-row scaling of VST values.


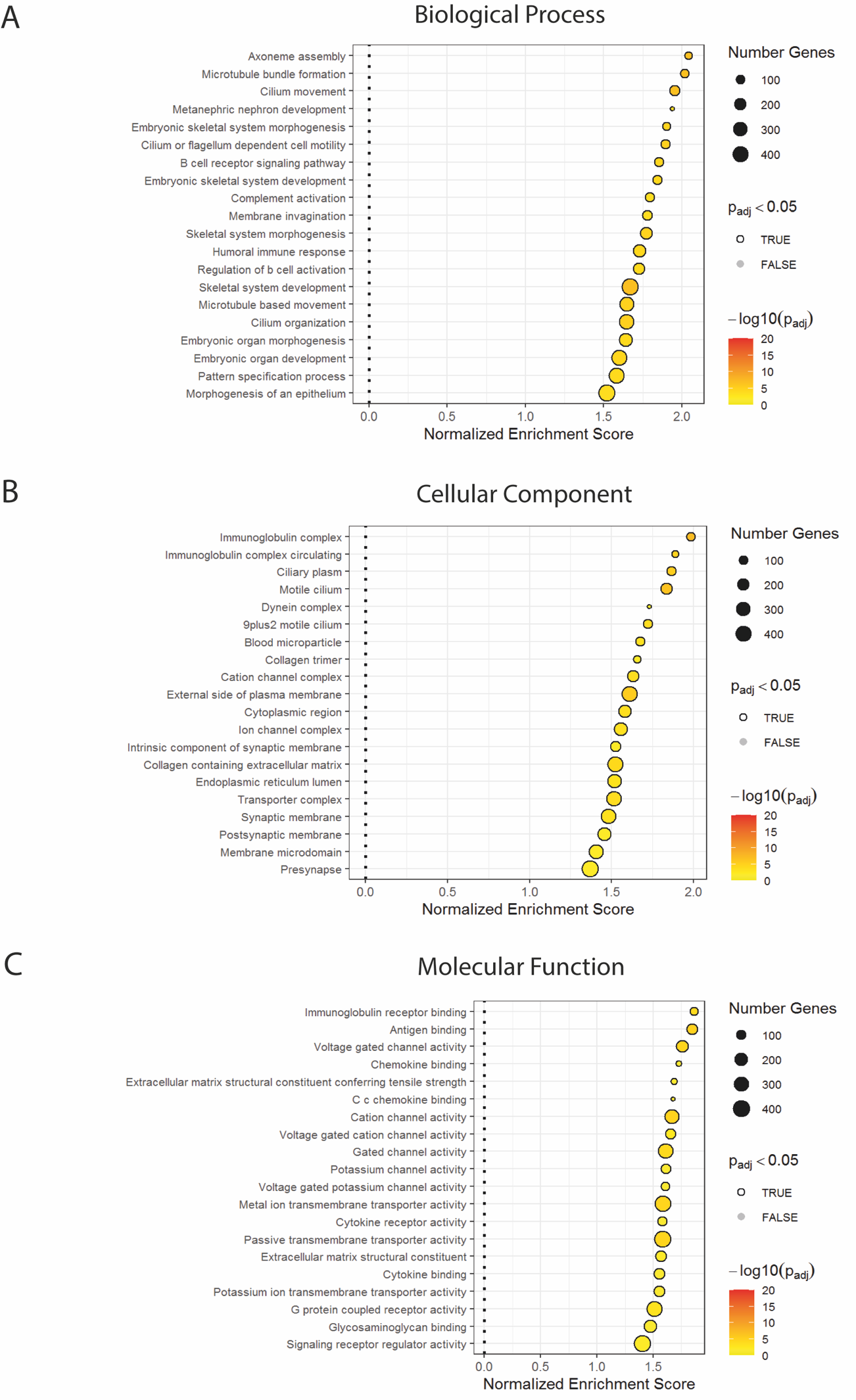


**Supplementary Figure 2. Gene ontologies of enriched differentially expressed genes between rh Bri2 BRICHOS R221E- and vehicle-treated WT mice.** Gene ontologies according to (A) biological processes, (B) cellular components, and (C) molecular functions of genes that are differentially expressed between rh Bri2 BRICHOS R221E- and vehicle-treated wild-type (WT) mice, shown in **Fig. 6C and D**.


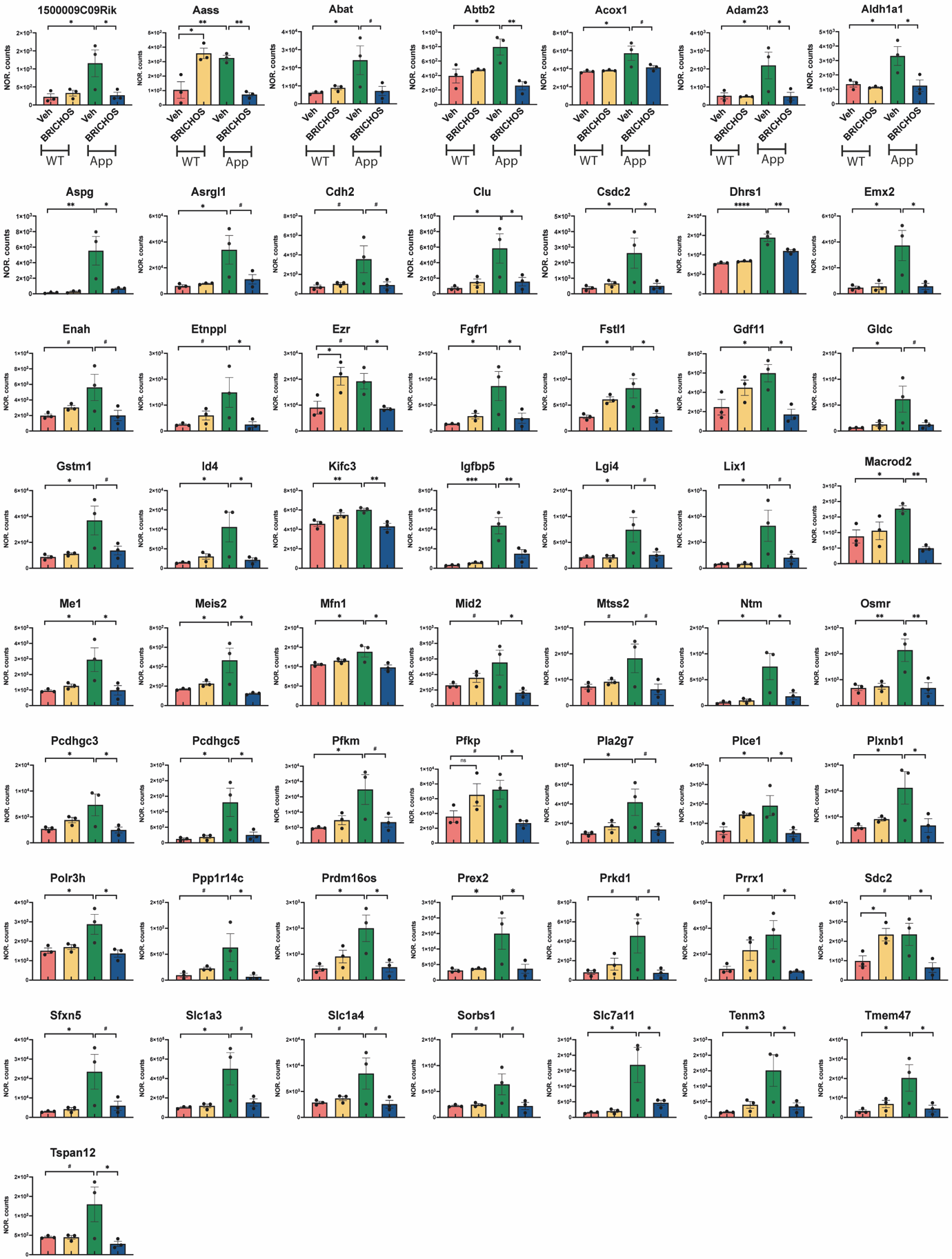


**Supplementary Figure 3. Genes with altered expression between WT and *App^NL-G-F^* mice that show normalization after rh Bri2 BRICHOS R221E treatment.** Normalized (NOR) counts for genes in **Fig. 7** that show *(i)* increased (p < 0.1) expression in *App^NL-G-F^* compared to WT mice, and *(ii)* altered expression in *App^NL-G-F^* mice treated with rh Bri2 BRICHOS. n = 3 mice/group. One-way ANOVA and Tukey’s *post hoc* test were used for statistical analysis. ∗p < 0.05, ∗∗p < 0.01, ∗∗∗p < 0.001, ∗∗∗∗p < 0.0001, #p <0.1.


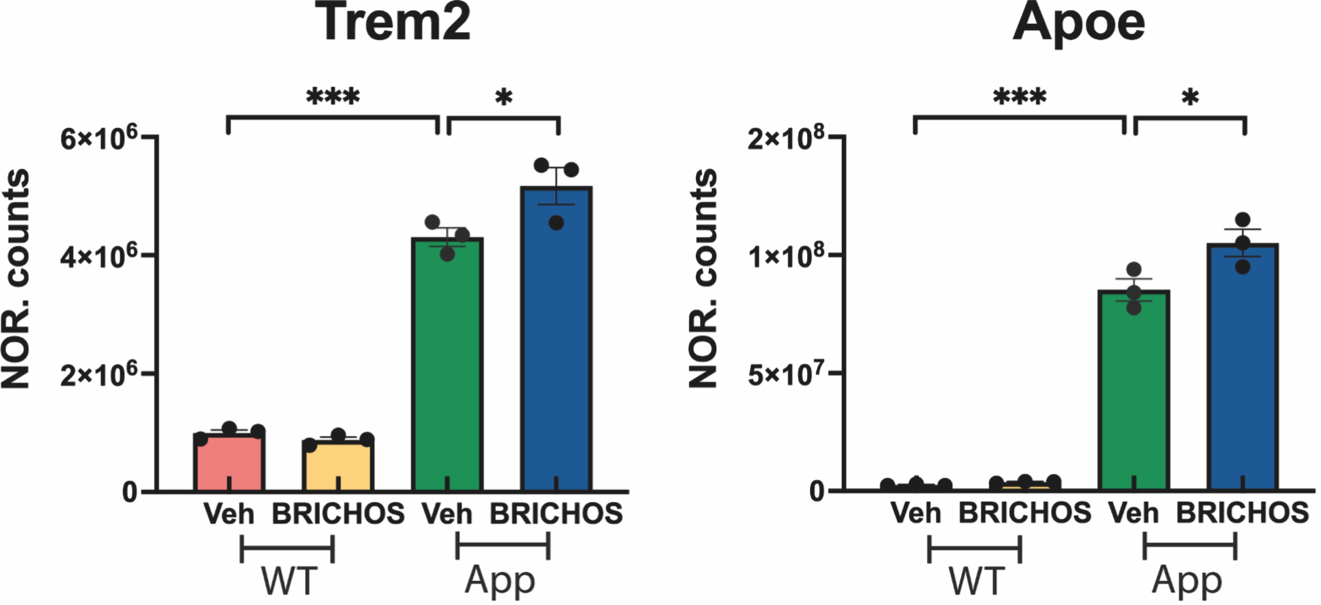


**Supplementary Figure 4. Expression of Trem2 and ApoE genes in WT and *App^NL-G-F^* mice.** Normalized (NOR) counts for *Trem2* and *ApoE* genes in the four treatment groups. n = 3 mice/group. One-way ANOVA and Tukey’s *post hoc* test were used for statistical analysis. ∗p < 0.05, ∗∗∗p < 0.001.


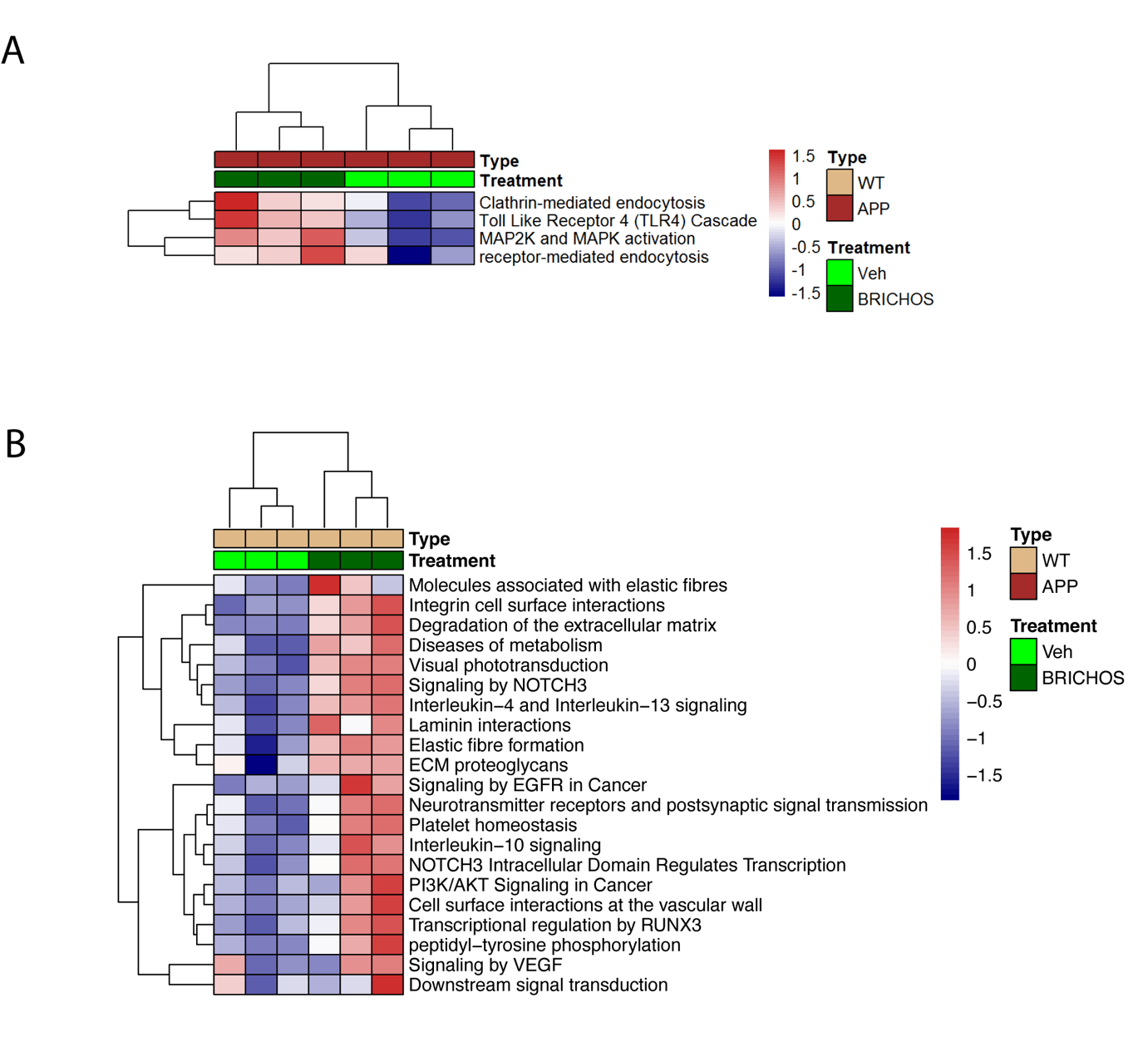


**Supplementary Figure 5A-B. Rh Bri2 BRICHOS R221E treatment effects on ligand-receptor pathways in *App^NL-G-F^* and WT mice.** Heatmap of significantly increased ligand-receptor biological pathways after rh Bri2 BRICHOS R221E or vehicle (Veh) treatment of *App^NL-G-F^* **(A)** and WT **(B)** mice. n = 3 mice/group. Heat coloring is shown as | log_2_fold change| >=1 and p< 0.05.

**Supplementary Table 1. Genes identified in Supplementary Figure 3**

| Gene ID | Encoded protein | AD/Aβ relevance Reference |
| --- | --- | --- |
| 1500009C09Rik | RIKEN cDNA 1500009C09 gene | - |
| Aass | Aminoadipate-semialdehyde synthase | - |
| Abat | 4-aminobutyrate aminotransferase | ^1, 2, 3, 4^ |
| Abtb2 | Ankyrin repeat and BTB domain containing 2 | - |
| Acox1 | Acyl-Coenzyme A oxidase 1, palmitoyl | - |
| Adam23 | Disintegrin and metalloproteinase domain-containing protein 23 | - |
| Aldh1a1 | Aldehyde dehydrogenase family 1, subfamily A1 | - |
| Aspg | Asparaginase | - |
| Asrgl1 | Asparaginase like 1 | - |
| Cdh2 | Cadherin 2 | ^5, 6, 7^ |
| Clu | Clusterin | ^8, 9, 10, 11, 12, 13, 14, 15^ |
| Csdc2 | Cold shock domain containing C2 | - |
| Dhrs1 | Dehydrogenase/reductase member 1 | - |
| Emx2 | Empty spiracles homeobox 2 | - |
| Enah | ENAH actin regulator | - |
| Etnppl | Ethanolamine phosphate phosphorylase | - |
| Ezr | Ezrin | ^16, 17^ |
| Fgfr1 | Fibroblast growth factor receptor 1 | ^18, 19, 20^ |
| Fstl1 | Follistatin-like 1 | ^21, 22, 23^ |
| Gdf11 | Growth differentiation factor 11 | - |
| Gldc | Glycine decarboxylase | - |
| Gstm1 | Glutathione S-transferase, mu 1 | ^24, 25, 26, 27, 28, 29^ |
| Id4 | Inhibitor of DNA binding 4 | - |

| Gene ID | Encoded protein | AD/Aβ AD/Aβ relevance Reference |
| --- | --- | --- |
| Igfbp5 | Insulin-like growth factor binding protein 5 | ^30, 31, 32^ |
| Kifc3 | Kinesin family member C3 | - |
| Lgi4 | Leucine-rich repeat LGI family, member 4 | - |
| Lix1 | Limb and CNS expressed 1 | - |
| Macrod2 | Mono-ADP ribosylhydrolase 2 | - |
| Me1 | Malic enzyme 1 | - |
| Meis2 | Meis homeobox 2 | - |
| Mfn1 | Mitofusin 1 | ^33, 34, 35^ |
| Mid2 | Midline 2 | - |
| Mtss2 | MTSS I-BAR domain containing 2 | ^36, 37^ |
| Ntm | Neurotrimin | - |
| Osmr | Oncostatin M receptor | - |
| Pcdhgc3 | Protocadherin gamma subfamily C, 3 | - |
| Pcdhgc5 | Protocadherin gamma subfamily C, 5 | - |
| Pfkm | Phosphofructokinase, muscle | - |
| Pfkp | Phosphofructokinase, platelet | ^38, 39^ |
| Pla2g7 | Phospholipase A2, group VII | ^40, 41, 42^ |
| Plce1 | Phospholipase C, epsilon 1 | ^43^ |
| Plxnb1 | Plexin B1 | ^44, 45^ |
| Polr3h | Polymerase (RNA) III (DNA directed) polypeptide H | - |
| Ppp1r14c | Protein phosphatase 1, regulatory inhibitor subunit 14C | - |
| Prdm16os | Prdm16 opposite strand transcript | - |
| Prex2 | Phosphatidylinositol-3,4,5-trisphosphate-dependent Rac exchange factor 2 | ^46, 47^ |
| Prkd1 | Protein kinase D1 | - |

| Gene ID | Encoded protein | AD/Aβ AD/Aβ relevance Reference |
| --- | --- | --- |
| Prrx1 | Paired related homeobox 1 | - |
| Sdc2 | Syndecan 2 | - |
| Sfxn5 | Sideroflexin 5 | - |
| Slc1a3 | Solute carrier family 1, member 3 | ^48, 49, 50, 51^ |
| Slc1a4 | Solute carrier family 1, member 4 | ^52, 53, 54, 55, 56^ |
| Slc7a11 | Solute carrier family 7 | ^57, 58, 59^ |
| Sorbs1 | Sorbin and SH3 domain containing 1 | ^60, 61, 62^ |
| Tenm3 | Teneurin transmembrane protein 3 | - |
| Tmem47 | Transmembrane protein 47 | - |
| Tspan12 | Tetraspanin 12 | ^63, 64, 65^ |

**Supplementary Table 2.** Primary antibodies used for immunohistochemistry

| **Protein targeted** | **Host** | **Dilution** | **Provider** | **Catalogue No.** |
| --- | --- | --- | --- | --- |
| Bri2 BRICHOS | Rabbit | 1:100 | Aviva systems biology | OAAB09465 |
| GFAP | Rabbit | 1:800 | Dako | Z033401-2;55769 |
| Iba1 | Rabbit | 1:400 | Wako | 019-19741 |
| Aβ (82E1 antibody) | Mouse | 1:200 | IBL | 10323 |

**Supplementary Table 3.** Secondary antibodies used for immunohistochemistry

| **Antibody** | **Host** | **Dilution** | **Provider** | **Catalogue No.** |
| --- | --- | --- | --- | --- |
| Alexa Fluor Plus 594 anti-mouse IgG | Donkey | 1:400 | Invitrogen | A32744 |
| Alexa Fluor Plus 488 anti-mouse IgG | Donkey | 1:400 | Invitrogen | A32766 |
| Swine Anti-Rabbit IgG | Pig | 1:200 | Dako | P0399 |

**Supplementary Excel file 1.** Lists of genes in Figure 6D.

**Supplementary Excel file 2.** Lists of genes in Figure 8.

**References**

1. Sherif FM, Ahmed SS. Basic aspects of GABA-transaminase in neuropsychiatric disorders. *Clin Biochem* **28**, 145-154 (1995).

2. Aoyagi T, Wada T, Nagai M, Kojima F, Harada S, Takeuchi T*, et al.* Increased gamma-aminobutyrate aminotransferase activity in brain of patients with Alzheimer's disease. *Chem Pharm Bull (Tokyo)* **38**, 1748-1749 (1990).

3. Sherif F, Gottfries CG, Alafuzoff I, Oreland L. Brain gamma-aminobutyrate aminotransferase (GABA-T) and monoamine oxidase (MAO) in patients with Alzheimer's disease. *J Neural Transm Park Dis Dement Sect* **4**, 227-240 (1992).

4. Zheng Q, Bi R, Xu M, Zhang DF, Tan LW, Lu YP*, et al.* Exploring the Genetic Association of the ABAT Gene with Alzheimer's Disease. *Mol Neurobiol* **58**, 1894-1903 (2021).

5. Andreyeva A, Nieweg K, Horstmann K, Klapper S, Muller-Schiffmann A, Korth C*, et al.* C-terminal fragment of N-cadherin accelerates synapse destabilization by amyloid-beta. *Brain* **135**, 2140-2154 (2012).

6. John A, Reddy PH. Synaptic basis of Alzheimer's disease: Focus on synaptic amyloid beta, P-tau and mitochondria. *Ageing Res Rev* **65**, 101208 (2021).

7. Choi JY, Cho SJ, Park JH, Yun SM, Jo C, Kim EJ*, et al.* Elevated Cerebrospinal Fluid and Plasma N-Cadherin in Alzheimer Disease. *J Neuropathol Exp Neurol* **79**, 484-492 (2020).

8. Harold D, Abraham R, Hollingworth P, Sims R, Gerrish A, Hamshere ML*, et al.* Genome-wide association study identifies variants at CLU and PICALM associated with Alzheimer's disease. *Nat Genet* **41**, 1088-1093 (2009).

9. Lambert JC, Heath S, Even G, Campion D, Sleegers K, Hiltunen M*, et al.* Genome-wide association study identifies variants at CLU and CR1 associated with Alzheimer's disease. *Nat Genet* **41**, 1094-1099 (2009).

10. Ghiso J, Matsubara E, Koudinov A, Choi-Miura NH, Tomita M, Wisniewski T*, et al.* The cerebrospinal-fluid soluble form of Alzheimer's amyloid beta is complexed to SP-40,40 (apolipoprotein J), an inhibitor of the complement membrane-attack complex. *Biochem J* **293 ( Pt 1)**, 27-30 (1993).

11. Matsubara E, Soto C, Governale S, Frangione B, Ghiso J. Apolipoprotein J and Alzheimer's amyloid beta solubility. *Biochem J* **316 ( Pt 2)**, 671-679 (1996).

12. Beeg M, Stravalaci M, Romeo M, Carra AD, Cagnotto A, Rossi A*, et al.* Clusterin Binds to Abeta1-42 Oligomers with High Affinity and Interferes with Peptide Aggregation by Inhibiting Primary and Secondary Nucleation. *J Biol Chem* **291**, 6958-6966 (2016).

13. Narayan P, Orte A, Clarke RW, Bolognesi B, Hook S, Ganzinger KA*, et al.* The extracellular chaperone clusterin sequesters oligomeric forms of the amyloid-beta(1-40) peptide. *Nat Struct Mol Biol* **19**, 79-83 (2011).

14. Boggs LN, Fuson KS, Baez M, Churgay L, McClure D, Becker G*, et al.* Clusterin (Apo J) protects against in vitro amyloid-beta (1-40) neurotoxicity. *J Neurochem* **67**, 1324-1327 (1996).

15. May PC, Johnson SA, Poirier J, Lampert-Etchells M, Finch CE. Altered gene expression in Alzheimer's disease brain tissue. *Can J Neurol Sci* **16**, 473-476 (1989).

16. Du Y, Bradshaw WJ, Leisner TM, Annor-Gyamfi JK, Qian K, Bashore FM*, et al.* Discovery of FERM domain protein-protein interaction inhibitors for MSN and CD44 as a potential therapeutic approach for Alzheimer's disease. *J Biol Chem* **299**, 105382 (2023).

17. Lian P, Cai X, Wang C, Liu K, Yang X, Wu Y*, et al.* Identification of metabolism-related subtypes and feature genes in Alzheimer's disease. *J Transl Med* **21**, 628 (2023).

18. Turner CA, Watson SJ, Akil H. The fibroblast growth factor family: neuromodulation of affective behavior. *Neuron* **76**, 160-174 (2012).

19. Qian K, Bao X, Li Y, Wang P, Guo Q, Yang P*, et al.* Cholinergic Neuron Targeting Nanosystem Delivering Hybrid Peptide for Combinatorial Mitochondrial Therapy in Alzheimer's Disease. *ACS Nano* **16**, 11455-11472 (2022).

20. Takami K, Matsuo A, Terai K, Walker DG, McGeer EG, McGeer PL. Fibroblast growth factor receptor-1 expression in the cortex and hippocampus in Alzheimer's disease. *Brain Res* **802**, 89-97 (1998).

21. Cheng KY, Liu Y, Han YG, Li JK, Jia JL, Chen B*, et al.* Follistatin-like protein 1 suppressed pro-inflammatory cytokines expression during neuroinflammation induced by lipopolysaccharide. *J Mol Histol* **48**, 63-72 (2017).

22. Dai D, Xie J, Zheng Y, Chen F, Zhao B, Miao L. H3K27 acetylation-induced FSTL1 upregulation by P300/RUNX1 co-activation exacerbated autophagy-mediated neuronal damage and NF-kappaB-stimulated inflammation in Alzheimer's disease. *Cytotechnology* **75**, 449-460 (2023).

23. Kumari E, Xu A, Chen R, Yan Y, Yang Z, Zhang T. FSTL1-knockdown improves neural oscillation via decreasing neuronal-inflammation regulating apoptosis in Aβ1–42 induced AD model mice. *Experimental Neurology* **359**, 114231 (2023).

24. Castellani R, Hirai K, Aliev G, Drew KL, Nunomura A, Takeda A*, et al.* Role of mitochondrial dysfunction in Alzheimer's disease. *J Neurosci Res* **70**, 357-360 (2002).

25. Cedazo-Minguez A, Cowburn RF. Apolipoprotein E: a major piece in the Alzheimer's disease puzzle. *J Cell Mol Med* **5**, 254-266 (2001).

26. Miranda S, Opazo C, Larrondo LF, Munoz FJ, Ruiz F, Leighton F*, et al.* The role of oxidative stress in the toxicity induced by amyloid beta-peptide in Alzheimer's disease. *Prog Neurobiol* **62**, 633-648 (2000).

27. Hayes JD, Strange RC. Glutathione S-transferase polymorphisms and their biological consequences. *Pharmacology* **61**, 154-166 (2000).

28. Lovell MA, Xie C, Markesbery WR. Decreased glutathione transferase activity in brain and ventricular fluid in Alzheimer's disease. *Neurology* **51**, 1562-1566 (1998).

29. Lin W, Zhang J, Liu Y, Wu R, Yang H, Hu X*, et al.* Studies on diagnostic biomarkers and therapeutic mechanism of Alzheimer's disease through metabolomics and hippocampal proteomics. *Eur J Pharm Sci* **105**, 119-126 (2017).

30. Rauskolb S, Andreska T, Fries S, von Collenberg CR, Blum R, Monoranu CM*, et al.* Insulin-like growth factor 5 associates with human Ass plaques and promotes cognitive impairment. *Acta Neuropathol Commun* **10**, 68 (2022).

31. Jin B, Cheng X, Fei G, Sang S, Zhong C. Identification of diagnostic biomarkers in Alzheimer's disease by integrated bioinformatic analysis and machine learning strategies. *Front Aging Neurosci* **15**, 1169620 (2023).

32. Foveau B, Correia AS, Hebert SS, Rainone S, Potvin O, Kergoat MJ*, et al.* Stem Cell-Derived Neurons as Cellular Models of Sporadic Alzheimer's Disease. *J Alzheimers Dis* **67**, 893-910 (2019).

33. Kerr JS, Adriaanse BA, Greig NH, Mattson MP, Cader MZ, Bohr VA*, et al.* Mitophagy and Alzheimer's Disease: Cellular and Molecular Mechanisms. *Trends Neurosci* **40**, 151-166 (2017).

34. Yan MH, Wang X, Zhu X. Mitochondrial defects and oxidative stress in Alzheimer disease and Parkinson disease. *Free Radic Biol Med* **62**, 90-101 (2013).

35. Wang X, Su B, Lee HG, Li X, Perry G, Smith MA*, et al.* Impaired balance of mitochondrial fission and fusion in Alzheimer's disease. *J Neurosci* **29**, 9090-9103 (2009).

36. Kesika P, Suganthy N, Sivamaruthi BS, Chaiyasut C. Role of gut-brain axis, gut microbial composition, and probiotic intervention in Alzheimer's disease. *Life Sci* **264**, 118627 (2021).

37. Adewuyi EO, O'Brien EK, Nyholt DR, Porter T, Laws SM. A large-scale genome-wide cross-trait analysis reveals shared genetic architecture between Alzheimer's disease and gastrointestinal tract disorders. *Commun Biol* **5**, 691 (2022).

38. Siddiqui T, Cosacak MI, Popova S, Bhattarai P, Yilmaz E, Lee AJ*, et al.* Nerve growth factor receptor (Ngfr) induces neurogenic plasticity by suppressing reactive astroglial Lcn2/Slc22a17 signaling in Alzheimer's disease. *NPJ Regen Med* **8**, 33 (2023).

39. Ma Y, Dammer EB, Felsky D, Duong DM, Klein HU, White CC*, et al.* Atlas of RNA editing events affecting protein expression in aged and Alzheimer's disease human brain tissue. *Nat Commun* **12**, 7035 (2021).

40. Schaloske RH, Dennis EA. The phospholipase A2 superfamily and its group numbering system. *Biochim Biophys Acta* **1761**, 1246-1259 (2006).

41. Acharya NK, Levin EC, Clifford PM, Han M, Tourtellotte R, Chamberlain D*, et al.* Diabetes and hypercholesterolemia increase blood-brain barrier permeability and brain amyloid deposition: beneficial effects of the LpPLA2 inhibitor darapladib. *J Alzheimers Dis* **35**, 179-198 (2013).

42. Maher-Edwards G, De'Ath J, Barnett C, Lavrov A, Lockhart A. A 24-week study to evaluate the effect of rilapladib on cognition and cerebrospinal fluid biomarkers of Alzheimer's disease. *Alzheimers Dement (N Y)* **1**, 131-140 (2015).

43. Hu D, Mo X, Jihang L, Huang C, Xie H, Jin L. Novel diagnostic biomarkers of oxidative stress, immunological characterization and experimental validation in Alzheimer's disease. *Aging (Albany NY)* **15**, 10389-10406 (2023).

44. Oinuma I, Ishikawa Y, Katoh H, Negishi M. The Semaphorin 4D receptor Plexin-B1 is a GTPase activating protein for R-Ras. *Science* **305**, 862-865 (2004).

45. Yu L, Petyuk VA, Gaiteri C, Mostafavi S, Young-Pearse T, Shah RC*, et al.* Targeted brain proteomics uncover multiple pathways to Alzheimer's dementia. *Ann Neurol* **84**, 78-88 (2018).

46. Xiong W, Cai J, Li R, Wen C, Tan H, On Behalf Of The Alzheimer's Disease Neuroimaging Initiative Adni D. Rare Variant Analysis and Molecular Dynamics Simulation in Alzheimer's Disease Identifies Exonic Variants in FLG. *Genes (Basel)* **13**, (2022).

47. Galea E, Weinstock LD, Larramona-Arcas R, Pybus AF, Gimenez-Llort L, Escartin C*, et al.* Multi-transcriptomic analysis points to early organelle dysfunction in human astrocytes in Alzheimer's disease. *Neurobiol Dis* **166**, 105655 (2022).

48. Kanai Y, Clemencon B, Simonin A, Leuenberger M, Lochner M, Weisstanner M*, et al.* The SLC1 high-affinity glutamate and neutral amino acid transporter family. *Mol Aspects Med* **34**, 108-120 (2013).

49. Cassano T, Serviddio G, Gaetani S, Romano A, Dipasquale P, Cianci S*, et al.* Glutamatergic alterations and mitochondrial impairment in a murine model of Alzheimer disease. *Neurobiol Aging* **33**, 1121 e1121-1112 (2012).

50. Tong H, Zhang X, Meng X, Xu P, Zou X, Qu S. Amyloid-beta peptide decreases expression and function of glutamate transporters in nervous system cells. *Int J Biochem Cell Biol* **85**, 75-84 (2017).

51. Chen S, Chang Y, Li L, Acosta D, Li Y, Guo Q*, et al.* Spatially resolved transcriptomics reveals genes associated with the vulnerability of middle temporal gyrus in Alzheimer's disease. *Acta Neuropathol Commun* **10**, 188 (2022).

52. Rosenberg D, Artoul S, Segal AC, Kolodney G, Radzishevsky I, Dikopoltsev E*, et al.* Neuronal D-serine and glycine release via the Asc-1 transporter regulates NMDA receptor-dependent synaptic activity. *J Neurosci* **33**, 3533-3544 (2013).

53. Sason H, Billard JM, Smith GP, Safory H, Neame S, Kaplan E*, et al.* Asc-1 Transporter Regulation of Synaptic Activity via the Tonic Release of d-Serine in the Forebrain. *Cereb Cortex* **27**, 1573-1587 (2017).

54. Traynelis SF, Wollmuth LP, McBain CJ, Menniti FS, Vance KM, Ogden KK*, et al.* Glutamate receptor ion channels: structure, regulation, and function. *Pharmacol Rev* **62**, 405-496 (2010).

55. Zhang Y, Li P, Feng J, Wu M. Dysfunction of NMDA receptors in Alzheimer's disease. *Neurol Sci* **37**, 1039-1047 (2016).

56. Puris E, Saveleva L, de Sousa Maciel I, Kanninen KM, Auriola S, Fricker G. Protein Expression of Amino Acid Transporters Is Altered in Isolated Cerebral Microvessels of 5xFAD Mouse Model of Alzheimer's Disease. *Mol Neurobiol* **60**, 732-748 (2023).

57. Lane HY, Lin CH. Diagnosing Alzheimer's Disease Specifically and Sensitively With pLG72 and Cystine/Glutamate Antiporter SLC7A11 AS Blood Biomarkers. *Int J Neuropsychopharmacol* **26**, 1-8 (2023).

58. Qin S, Colin C, Hinners I, Gervais A, Cheret C, Mallat M. System Xc- and apolipoprotein E expressed by microglia have opposite effects on the neurotoxicity of amyloid-beta peptide 1-40. *J Neurosci* **26**, 3345-3356 (2006).

59. Schallier A, Smolders I, Van Dam D, Loyens E, De Deyn PP, Michotte A*, et al.* Region- and age-specific changes in glutamate transport in the AbetaPP23 mouse model for Alzheimer's disease. *J Alzheimers Dis* **24**, 287-300 (2011).

60. Barbagallo M, Dominguez LJ. Type 2 diabetes mellitus and Alzheimer's disease. *World J Diabetes* **5**, 889-893 (2014).

61. Hayden MR. Type 2 Diabetes Mellitus Increases The Risk of Late-Onset Alzheimer's Disease: Ultrastructural Remodeling of the Neurovascular Unit and Diabetic Gliopathy. *Brain Sci* **9**, (2019).

62. Blalock EM, Geddes JW, Chen KC, Porter NM, Markesbery WR, Landfield PW. Incipient Alzheimer's disease: Microarray correlation analyses reveal major transcriptional and tumor suppressor responses. *P Natl Acad Sci USA* **101**, 2173-2178 (2004).

63. Seipold L, Saftig P. The Emerging Role of Tetraspanins in the Proteolytic Processing of the Amyloid Precursor Protein. *Front Mol Neurosci* **9**, 149 (2016).

64. Postina R, Schroeder A, Dewachter I, Bohl J, Schmitt U, Kojro E*, et al.* A disintegrin-metalloproteinase prevents amyloid plaque formation and hippocampal defects in an Alzheimer disease mouse model. *J Clin Invest* **113**, 1456-1464 (2004).

65. Xu D, Sharma C, Hemler ME. Tetraspanin12 regulates ADAM10-dependent cleavage of amyloid precursor protein. *FASEB J* **23**, 3674-3681 (2009).
